# Taurine Electrografting onto Porous Electrodes Improves Redox Flow Battery Performance

**DOI:** 10.1021/acsami.2c08211

**Published:** 2022-09-07

**Authors:** Emre B. Boz, Pierre Boillat, Antoni Forner-Cuenca

**Affiliations:** † Electrochemical Materials and Systems, Department of Chemical Engineering and Chemistry, Eindhoven University of Technology, P.O. Box 513, 5600 MB Eindhoven, The Netherlands; ‡ Eindhoven Institute for Renewable Energy Systems, Eindhoven University of Technology, P.O. Box 513, 5600 MB Eindhoven, The Netherlands; § Electrochemistry Laboratory, Paul Scherrer Institute, Forschungsstrasse 111, CH-5232 Villigen, Switzerland; ∥ Laboratory for Neutron Scattering and Imaging, Paul Scherrer Institute, Forschungsstrasse 111, CH-5232, Villigen, Switzerland

**Keywords:** electrografting, taurine, porous carbon electrodes, redox flow batteries, neutron radiography, wettability, energy storage

## Abstract

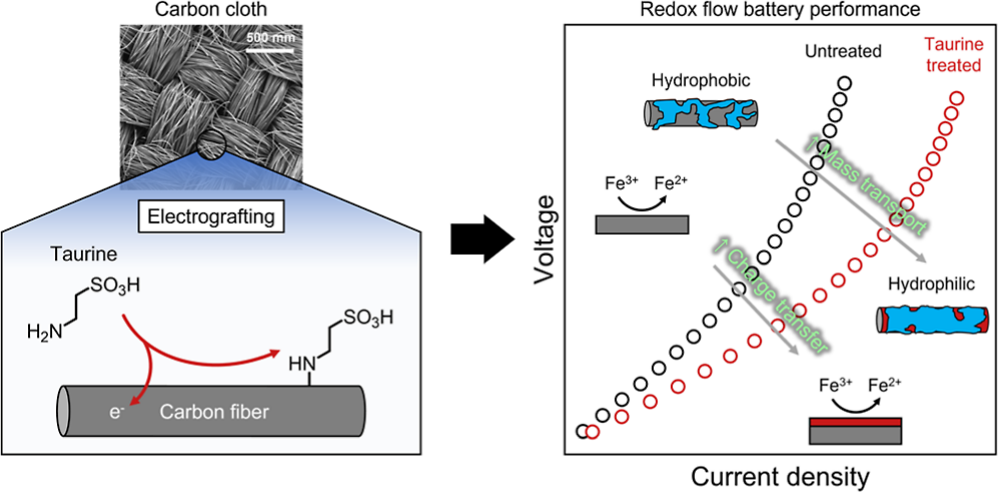

The surface properties of porous carbonaceous electrodes govern the performance, durability, and ultimately the cost of redox flow batteries (RFBs). State-of-the-art carbon fiber-based electrode interfaces suffer from limited kinetic activity and incomplete wettability, fundamentally limiting the performance. Surface treatments for electrodes such as thermal and acid activation are a common practice to make them more suitable for aqueous RFBs; however, these treatments offer limited control over the desired functional properties. Here, we propose, for the first time, electrografting as a facile, rapid, and versatile technique to enable task-specific functionalization of porous carbonaceous electrodes for use in RFBs. Electrografting allows covalent attachment of organic molecules on conductive substrates upon application of an electrochemical driving force, and the vast library of available organic molecules can unlock a broad range of desired functional properties. To showcase the potential of electrografting for RFBs, we elect to investigate taurine, an amine with a highly hydrophilic sulfonic acid tail. Oxidative electrografting with cyclic voltammetry allows covalent attachment of taurine through the amine group to the fiber surface, resulting in taurine-functionalized carbon cloth electrodes. In situ polarization and impedance spectroscopy in single-electrolyte flow cells reveal that taurine-treated cloth electrodes result in 40% lower charge transfer and 25% lower mass transfer resistances than off-the-shelf cloth electrodes. We find that taurine-treated electrode interfaces promote faster Fe^3+^ reduction reaction kinetics as the electrochemical surface area normalized current densities are 2-fold and 4-fold higher than oxidized and untreated glassy carbon surfaces, respectively. Improved mass transfer of taurine-treated electrodes is attributed to their superior wettability, as revealed by operando neutron radiography within a flow cell setup. Through demonstrating promising results for aqueous systems with the model molecule taurine, this work aims to bring forth electrografting as a facile technique to tailor electrode surfaces for other RFB chemistries and electrochemical technologies.

## Introduction

Grid-scale energy storage is poised to play a central role in the integration of intermittent renewable sources such as wind and solar. Among the energy storage options, redox flow batteries (RFBs) have emerged as promising technological candidates to level the discrepancies between supply and demand.^1,2^ RFBs are a class of secondary batteries where the active species dissolved in liquid electrolytes are stored in external tanks and pumped through an electrochemical stack where the redox reactions take place. Their simple design enables long operation lifetimes and allows decoupling of the power and energy units,^2−5^ making this technology suitable for grid-scale energy storage. However, current RFB constructs are not cost competitive for widespread implementation, motivating research into new electrolyte chemistries and advanced reactor designs.^6^ Irrespective of the flow battery chemistry, porous electrodes are performance-defining components as they provide the active sites for the redox reactions, the flow paths for the liquid electrolyte, and the electronic conductivity through the solid phase. Thus, the electrode microstructure determines the mass transport characteristics and pressure drop, whereas its surface chemical state and electrochemically active surface area govern the reaction rate, directly influencing the final stack costs of RFBs.^7−9^

State-of-the-art flow batteries leverage commercially available porous carbonaceous electrodes (e.g., felts, papers, and cloths), based on fiber-based mats in sheet-like arrangements, which are generally repurposed from more mature technologies such as low-temperature fuel cells.^9,10^ Also, while this technological spinout has enabled rapid progress due to knowledge and infrastructure reuse, employing these carbonaceous electrodes in flow battery reactors brings forth unique challenges. For example, the hydrophobic properties of carbon fiber-based porous electrodes challenge the operation of aqueous systems as capillary forces prevent full wetting of the electrode.^11^ Furthermore, the relative inertness of the carbon surface toward aqueous metal redox couples (V^2+/3+^ and Fe^2+/3+^) is another drawback of pristine carbonaceous materials, limiting electrochemical kinetics and resulting in cell overpotentials.^12,13^ Large charging potentials and prolonged cycling can modify the surface chemistry of the carbon electrode, resulting in decreased cell performance.^14,15^ Thus, engineering electrode interfaces to sustain the stringent requirements of liquid phase electrochemistry is a powerful strategy to reduce stack costs.

To address these challenges, various surface treatment strategies, such as thermal,^16,17^ acid treatments,^18^ anodic oxidation,^19,20^ and nanomaterial deposition,^21−24^ have been implemented with varying degrees of success. Most of these strategies aim to increase the heteroatom content of carbon surfaces as heteroatoms impact the local electronic structure by creating polar sites, which influence the surface energy and reactivity.^25^ Currently, the industry standard is the heat treatment of carbon fiber substrates, which induces surface oxidation by exposing the fiber surface to air or an oxygen-enriched atmosphere at high temperatures (400–500 °C). Among other desired effects, the heat treatment causes degradation of the fiber surface and mass loss, followed by embrittlement of the material at long treatment times.^26^ In fact, other oxidative methods such as acid and anodic treatments can degrade the substrate as well.^20,27^ While existing methods are effective for some redox chemistries, their application offers limited control over the nature and amount of the functional groups, and as a consequence, the resulting functional properties. We envision that the surface functionalities relevant for RFB electrodes can range from a catalytically active layer to boost reaction rates, a wettability-enhancing layer to ensure effective use of electrode area, or an inhibiting layer to prevent side reactions (e.g., hydrogen evolution).^28,29^ Consequently, the electrode treatment strategies should offer a broad design space by selecting and tuning the surface functionality with exceptional molecular specificity.

Here, we report, for the first time, electrografting as a surface modification strategy for RFB electrodes. With electrografting, covalently attached organic layers can be grown on porous carbonaceous electrodes and address the unique requirements of RFBs mentioned before. Electrografting is the electrochemical analogue of chemical grafting where organic molecules form covalent bonds with the electrode surface.^30^ It has advantages over chemical grafting as molecule-specific modification of the surface is possible without harsh treatment steps (e.g., strong acids/bases and high temperature) or dangerous chemicals (e.g., acyl chlorides) as reactive intermediates are created only in the near-surface region of the electrode and subsequently attack the electrode surface. In contrast to traditional coating methods such as dip coating or spraying, electrografting allows functionalization of the porous electrodes in a highly conformal fashion.^31^ Electrodes modified by electrografting harbor immobilized organic molecules on their surface that modulate the interfacial properties of the electrode. These organic molecules can be selected from a vast library of amines, diazonium salts, alcohols, or acids bearing various functional groups.^30^ In this work, we decided to graft a model amino acid and a physiologically important biomolecule, taurine, as it has an amine group on one end and a sulfonic acid group on the other end. Sulfonic acid is a functional group with high acidity and good water solubility and has been investigated as a surface group for electrodes in aqueous RFBs.^32−34^ Furthermore, amines are promising for electrografting as their electrochemistry is well studied, they bond covalently with many surfaces, and they can be molecularly engineered with various functional groups.

Electrografting of amines onto carbon fibers has been proposed to facilitate contact between epoxy and carbon fibers in composite materials.^35^ Since then, it has been revealed that anodic grafting of primary amines proceeds through oxidation to a radical cation, then generation of a neutral aminyl radical, and its subsequent attack on the substrate.^36^ Thus, as one of the simplest amines with a sulfonic acid group, taurine is a promising model molecule to utilize in electrografting reactions to create hydrophilic interfaces. There are previous reports on taurine electrografting mainly for sensor applications and the final layer is sometimes referred to as poly(taurine).^37−44^ We decided to avoid this designation as we could not unambiguously determine if taurine forms a polymeric layer. The taurine coating in previous studies has been used for the detection of neurotransmitters,^37^ pharmaceutical compounds,^38−40^ immobilization of enzymes,^41,43^ or to improve the oxidation of species on the carbon surface.^44^ The underlying mechanism could be based on a surface charge or a preconcentration effect as the coating can effectively block negatively charged species and increase the response of positively charged ones at low concentrations.^42^ However, the application of taurine electrografting has not been studied yet in the field of RFBs.

In this paper, we first study the electrochemical response of carbon electrodes during the electrografting reaction of taurine. Second, we employ X-ray photoelectron spectroscopy to reveal the degree of electrode functionalization and the chemical nature of the electrografted layer on cloth electrodes. Third, we determine the Fe^3+^ reduction kinetic rates on functionalized glassy carbon electrodes by hydrodynamic voltammetry. Fourth, we perform single-electrolyte flow cell studies in a mixed Fe^2+/3+^ electrolyte to understand the influence of the electrografted taurine layer on the different resistances of the cloth electrodes. Finally, we visualize electrode wettability within a flow cell with neutron radiography for the first time. We envision that the findings from this work can open a new path for task-specific electrode functionalization for RFBs and other electrode-driven electrochemical technologies.

## Materials and Methods

### Chemicals

Sodium chloride (NaCl, Sanal P+, >99%), potassium chloride (KCl, Sigma-Aldrich, 99%), sodium phosphate dibasic (Na_2_HPO_4_, Sigma-Aldrich, ≥98.5%), potassium phosphate monobasic (KH_2_PO_4_, Sigma-Aldrich, ≥99%), hydrochloric acid (HCl, Sigma-Aldrich, 37%), sodium hydroxide (NaOH, Merck, 1 M), taurine (2-aminoethanesulfonic acid, Sigma-Aldrich, ≥99%), iron(III) chloride hexahydrate (FeCl_3_·6H_2_O, Sigma-Aldrich, ≥99%), and iron(II) chloride tetrahydrate (FeCl_2_·4H_2_O, Sigma-Aldrich, ≥99.0%) were used without further purification. Ultrapure water was used in every experiment (18.2 MΩ, ELGA PURELAB).

### Electrografting

Phosphate-buffered saline (PBS, pH = 7.4, 0.1 M) solution was prepared according to the formula; 8 g of NaCl, 200 mg of KCl, 1.44 g of Na_2_HPO_4_, and 254 mg of KH_2_PO_4_ were dissolved in 1 L of ultrapure water and adjusted to pH = 7.4 with a 1 M HCl or 1 M NaOH solution. Taurine was dissolved in PBS solution to 0.1 M concentration, and the final pH was adjusted to 7.4 with a 1 M NaOH solution. Electrografting experiments were carried out in a glass electrochemistry cell (Metrohm AG) filled with 100 mL of a 0.1 M taurine solution (in 0.1 M PBS).

Glassy carbon and porous carbon cloth electrodes were used as substrates for electrografting. A glassy carbon electrode (GCE) in a rotating-disk electrode (RDE) form (Pine Instruments) has a surface area of 0.19634 cm^2^. Prior to immersion into the electrolyte, the GCE was polished to a mirror finish with 0.05 μm alumina slurry on a polishing pad. The carbon cloth (Nuvant ELAT Hydrophilic, Fuel Cell Store) has a nominal thickness of 0.406 mm, and an approximately 2 cm × 2 cm piece (≈4 cm^2^) was immersed in the electrolyte for the electrografting experiments. A 1.5 cm × 1.7 cm piece (2.55 cm^2^) was cut carefully from this piece to use as the electrode for the flow cell experiments. The electrografting substrate, Pt mesh (Pt, 99%, 2 cm diameter), and silver–silver chloride (BASi, Ag/AgCl in 3 M KCl) electrodes were used in a three-electrode setup as the working, counter, and reference electrodes, respectively. A Pt mesh was flame-annealed before each experiment to eliminate possible contamination from organics. Electrografting experiments were conducted via cyclic voltammetry (CV) mode of the potentiostat (Biologic VMP-300) where the potential was swept from −1.5 to 1.8 V (vs Ag/AgCl) 10 times with automatic iR compensation (solution resistance extracted at 100 kHz and 20 mV sinus amplitude, 85% compensation applied).

### X-ray Photoelectron Spectroscopy

The surface functionalities were determined using a Thermo Scientific K-alpha X-ray photoelectron spectrometer equipped with a monochromatic small-spot X-ray source and a 180° double focusing hemispherical analyzer with a 128-channel detector. An aluminum anode (Al Kα = 1486.6 eV) source operating at 72 W and a spot size of 400 μm were used to obtain the spectra. Survey scans were measured at a constant pass energy of 200 eV and region scans were measured at 50 eV. The background pressure was set to 2 × 10^–8^ mbar and, during the measurement, 4 × 10^–7^ mbar argon because of the charge compensation. For etching experiments, samples were exposed to 1 keV Ar^+^ ions with a >1 μA beam current, directed from an EX06 Ion Source situated within the XPS chamber. The etch area was approximately 5 times the X-ray spot size. Samples were exposed to the ion beam for a minute, after which a snapshot of the S 2p peak was recorded. This sequence was repeated 50 times or until the signal reached the background levels.

### Hydrodynamic Voltammetry

The hydrodynamic voltammetry experiments were carried out in 0.2 M Fe^3+^ in 2 M HCl with automatic iR compensation. The linear-sweep voltammetry (LSV) mode of the potentiostat (Biologic VMP-300) was used to sweep the potential cathodically from 1 to −0.6 V (vs Ag/AgCl) under 100, 400, 900, and 1600 rpm rotation of the RDE. Experiments were also carried out under exact conditions in the absence of Fe^3+^ to extract the background current of the electrodes, and all RDE polarization curves were background-corrected before further analysis.

Mass transfer effects were corrected by extrapolating the reciprocal of current to an infinite rotation rate to extract the kinetic current. The resulting kinetic currents were plotted against their respective potentials to draw a Tafel plot.^45^ Extrapolation of the Tafel plot to zero overpotential (taken as 0.5 V vs Ag/AgCl, which is close to 0.7 V vs the normal hydrogen electrode in 1 M HCl as reported previously)^45^ for the studied reaction gives the exchange current according to eq 1, from which the kinetic rate constant can be extracted.
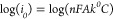
1where  is the exchange current (mA),  is the number of electrons transferred (1 in this case),  is the Faraday constant (96,485 C mol^–1^),  is the electrode area (0.19634 cm^2^),  is the kinetic rate constant (cm s^–1^), and  is the bulk concentration of the analyte (0.2 M). From the Tafel slope, the transfer coefficient () can be calculated with eq 2.
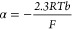
2where  is the Tafel slope (V^–1^) and the rest of the symbols have their usual significance.

### Capacitance Measurements

The capacitance of the electrodes within the flow cells was measured using CV with a 2 M HCl electroyte and at ∼5 cm s^–1^ velocity by cycling the potential from −0.2 to 0.2 V at varying scan rates (5–20–50–100–200 mV s^–1^). The capacitance of GCEs was measured by CV mode in 0.1 M KCl with no stirring and by cycling the potential from 0 to 0.4 V (vs Ag/AgCl) at the same scan rates in a three-electrode setup. All measurements were corrected for solution resistance by automatic iR compensation as described before. The current values were extracted for anodic and cathodic sweep and the differential capacitance of cells was calculated by eq 3.
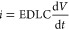
3where  is the average current in absolute value (mA) at mid-voltage value of the scan bounds,  is the electric double-layer capacitance (F), and  is the scan rate (mV s^–1^). The electrochemical surface area (ECSA) of the electrodes can then be calculated with eq 4.
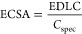
4where  is the electrochemical surface area (m^2^) and  is the area-specific capacitance of carbon materials taken as 23 μF cm^–2^.^46,47^ The ECSA can be normalized to the geometric surface area which then gives a roughness value () by eq 5.
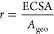
5where  (m^2^) is the geometric surface area, which is 0.07 cm^2^ for the GCE.

### Flow Cell Tests

#### Cell Setup

Single-electrolyte flow cell experiments (polarization, impedance, and stability measurements) were conducted with a redox flow cell.^48^ The flow diffusers of the cell were machined from polypropylene (McMaster-Carr) and the graphite flow-through flow fields, also acting as current collectors, were milled from 3.18 mm thick resin-impregnated graphite (G347B graphite, MWI, Inc.) and had one inlet channel and 1.5 cm of channel length. Both anode and cathode compartments housed one cloth electrode of the same type. A Nafion 211 membrane was soaked in 2 M HCl overnight and was placed between the two identical electrodes. Incompressible PTFE gaskets were used to seal the electrode area with the dimensions of 1.7 by 1.5 cm (2.55 cm^2^ geometric area). The electrodes were compressed by applying 2 N m torque, and the final compression was set to ∼20% by adjusting the gasket thickness. After ∼30 min, the cells were recompressed at the same torque to account for mechanical relaxation of the cell body. For the electrolyte, 0.1 M FeCl_3_·6H_2_O and 0.1 M FeCl_2_·4H_2_O were dissolved in 2 M HCl. The flow rate was maintained by a peristaltic pump (Cole-Parmer) and circulated using rubber tubes (Masterflex) through the cell. The electrolyte flow was adjusted to reach superficial velocities of 0.5, 1.5, 5.0, and 20.0 cm s^–1^ as calculated with eq 6.
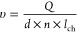
6where  is the electrolyte velocity (cm s^–1^),  is the flow rate (mL s^–1^),  is the compressed thickness of the electrode (0.033 cm),  is the number of inlet channels, and  is the channel length of the flow field. Pressure drop measurements were conducted with water at varying flow rates, and the average of inlet–outlet pressure difference was recorded.

#### Electrochemical Conditions

Electrochemical measurements for the flow cells were conducted using a Biologic VSP-3e potentiostat. Cells were subjected to preconditioning for three cycles from 0 to 0.2 V versus OCV at 20.0 cm s^–1^. For polarization measurements and preconditioning cycles, the cells were subjected to a staircase potential waveform with 25 mV steps, each lasting 60 s. The median value of the last 50 current values was considered as the current value for each step to account for steady-state conditions. For polarization measurements, the cells were polarized until the potentiostat current output limit was reached (1.2 A) or until a 0.6 V (vs OCV) threshold to prevent oxidation of the electrodes and current collectors at mass transfer limited conditions. Impedance spectroscopy was performed at OCV, from 200 kHz to 10 mHz at 20 mV sinus amplitude with eight measurement points per decade. Polarization and impedance measurements were repeated at each velocity in the decreasing order of 20.0–5.0–1.5–0.5 cm s^–1^. The polarization curves were compensated for the nondistributed area-specific ohmic resistance by correcting for the ohmic resistance value obtained from fitting the impedance spectra with an equivalent circuit.^48^

### Neutron Radiography

Neutron radiography was performed in the NEUTRA thermal neutron radiography beamline of the SINQ facility at the Paul Scherrer Institute, Switzerland. After being ejected from a lead spallation target, the neutrons are moderated to thermal velocities and a neutron beam is guided to the beamline with a mean energy of 25 meV and with a flux of 9.8 × 10^6^ neutrons cm^–2^ s^–1^ mA^–1^. After passing through the flow cell, the attenuated beam hits the detector and an image is formed on the scintillator screen. The tilted detector setup enables imaging of the zero-gap membrane electrode assembly by stretching the image in the horizontal transverse direction (with respect to beam trajectory).^49^ The flow cells used in neutron imaging utilized flow-by flow fields and a dense PTFE separator to reduce the influence of the membrane and flow field on electrode wetting. Flow-by flow fields were milled from 3.18 mm thick resin-impregnated graphite (G347B graphite, MWI, Inc.) and featured seven inlet channels with 1 mm width and 1.5 cm channel length. Equation 6 is modified to take into account channel depth (0.5 mm) instead of electrode thickness, and channel width (1 mm) instead of channel length in order to calculate the electrolyte velocity within the channels of the flow-by flow field. As the electrolyte solution, 1 M HCl in ultrapure water (nondeuterated) is used to maximize the contrast between the wetting and the nonwetting phase. The details of the image processing can be found elsewhere.^50,51^ In brief, all generated images were corrected for the spatial intensity variation in the beam (open beam correction), noise of the CCD detector when the beam is off (dark current correction), and spatial variation in the intensity due to scattered background radiation which depends on the sample type and geometry (black-body correction). A reference image was made by placing the flow cell without any liquid in the beamline; this was named the dry cell image. When the images of the wet cell were referenced by the dry cell, it is possible to quantify the electrolyte in the cell without any interference from the cell parts. Then, the attenuation of the beam by the electrolyte was calculated according to the Lambert–Beer law in eq 7.
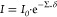
7where  is the attenuated intensity,  is equal to 1 since the images are corrected with respect to the dry cell (where the beam is not attenuated by liquid), and  is the macroscopic cross section of the electrolyte (cm^–1^), which was taken as 0.35 mm^–1^ for the NEUTRA beamline for nondeuterated water, and  is the thickness of the electrolyte (cm).^51^ Although hydrogen and chlorine have similar attenuation coefficients,^50^ the total attenuation caused by the chlorine ions is negligible as the concentration of hydrogen atoms is far greater in an aqueous solution of 1 M HCl. The saturation of the electrode was calculated using eq 8.
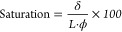
8where  is the thickness of the electrode (cm) in the beam direction, which is 1.5 cm, and  is the porosity of the cloth electrode, which is 80% according to the manufacturer.^52^

## Results and Discussion

### Taurine Electrografting

**Scheme 1:**
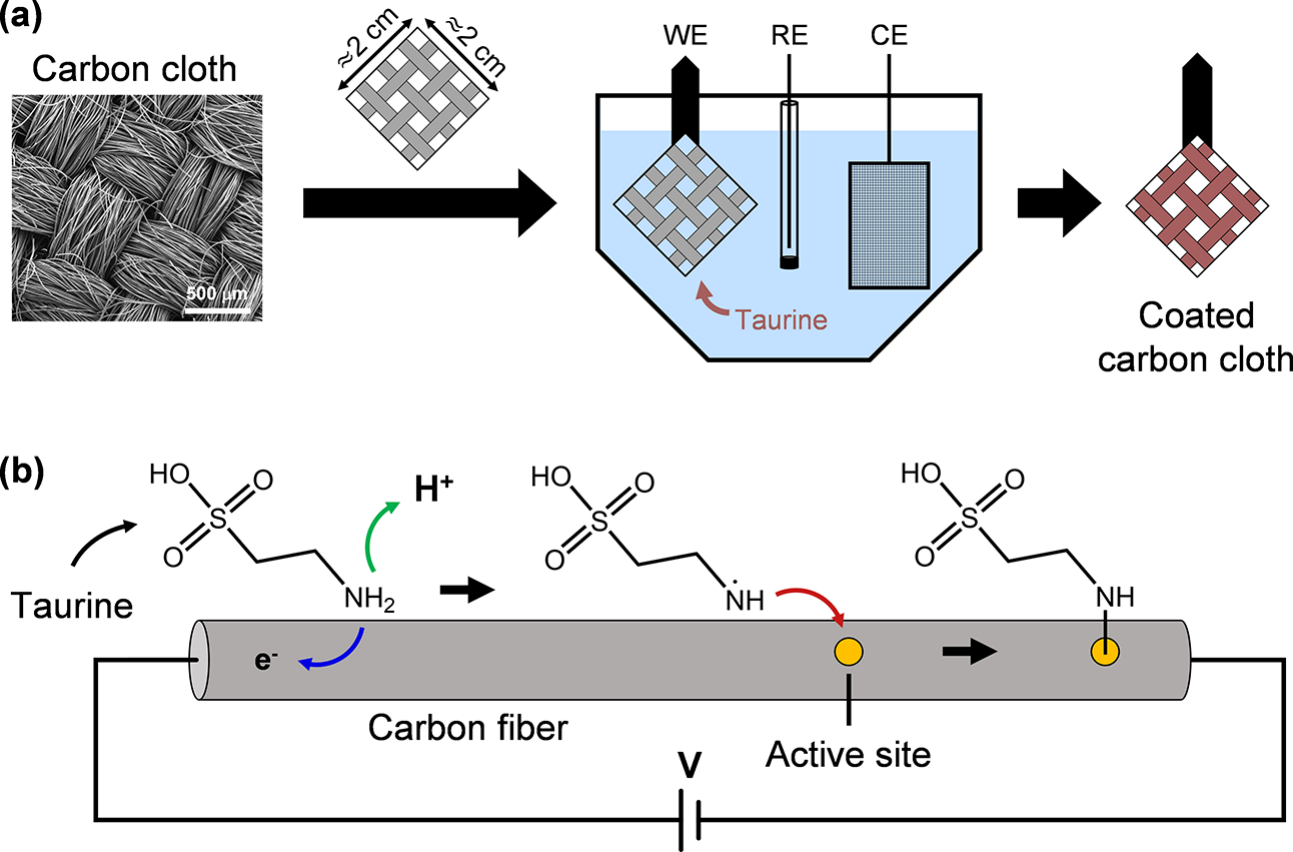
Illustration of the Taurine Electrografting Process on Porous Electrodes (a) Scheme of the electrografting where the working electrode is a porous carbon cloth cut in shape, the reaction potential is controlled with the reference electrode (RE), and the circuit is completed with the counter electrode (CE); (b) reaction mechanism of taurine electrografting where oxidation of the amine end results in generation of the aminyl radical which attacks the carbon surface, possibly on an active site.

We first study the electrografting reaction of taurine on carbon electrodes. The general methodology of electrografting of taurine on porous electrodes can be seen in Scheme 1a. The amine group of taurine can be oxidatively electrografted as seen in Scheme 1b, whereas the hydrophilic sulfonic acid group increases the wettability of the electrodes. We hypothesize that, based on previous studies that leveraged sulfonic acid functionalization, this functional group also improves the aqueous RFB performance.^32−34^

**Figure 1:**
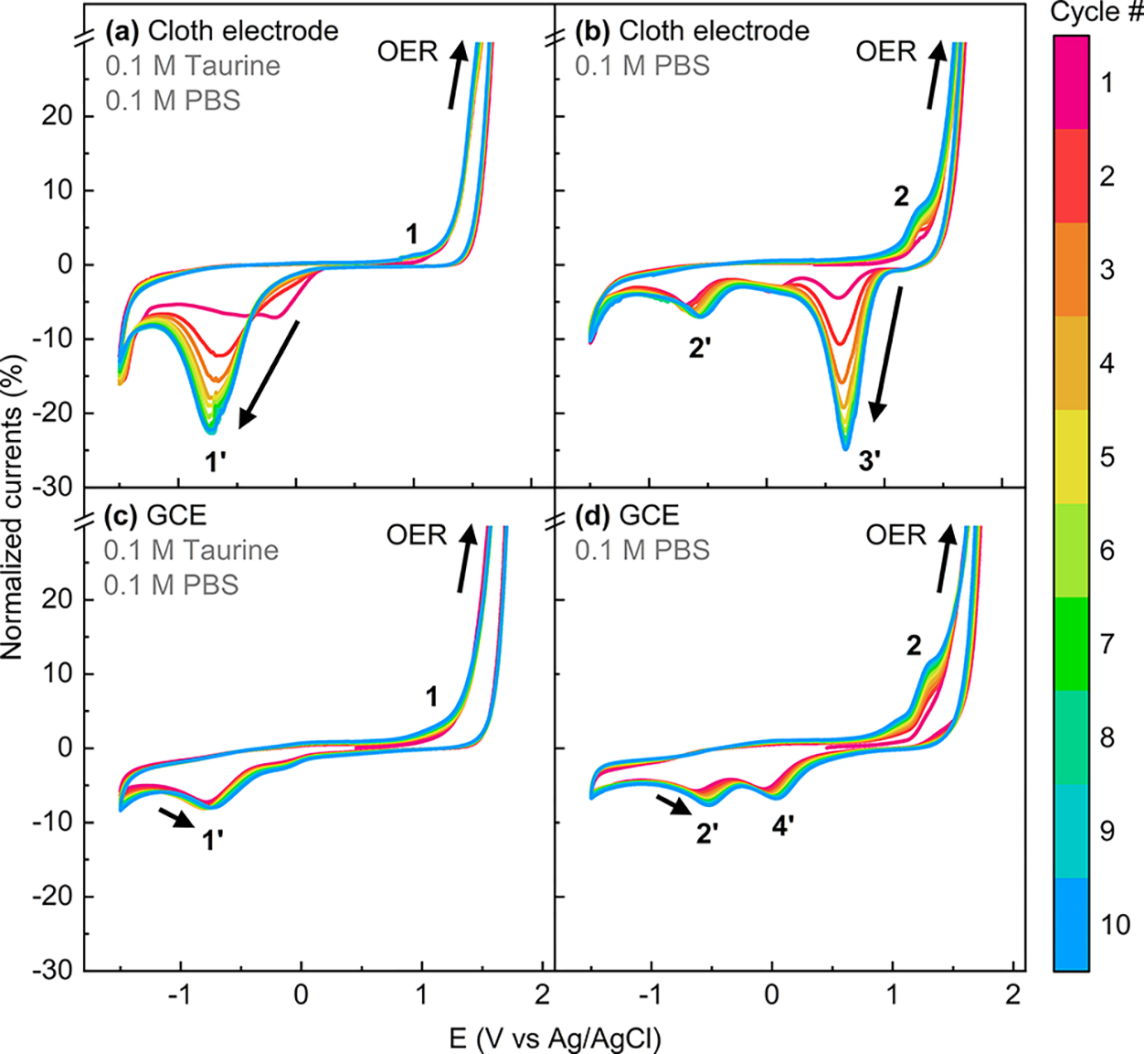
CV of carbon electrodes under various electrolytes, with and without taurine. (a) CV response of a cloth electrode in 0.1 M taurine + 0.1 M PBS and (b) in 0.1 M PBS. (c) CV response of the GCE in 0.1 M taurine + 0.1 M PBS and (d) in 0.1 M PBS. The OER peak has been cut for clarity.

Following the reported procedures, the potential is cycled between −1.5 to 1.8 V (vs Ag/AgCl) 10 times in 0.1 M taurine and 0.1 M PBS, which is used as the pH buffer.^37−41,43,44^ We opted for a higher concentration of taurine compared to the literature to account for the larger surface area of the carbon cloth electrodes. The electrochemical response of the cloth electrode in the taurine + PBS electrolyte can be seen in Figure 1a. Here, the main peaks are a large oxidation peak at the anodic limit of the CV and a reduction peak centered at −0.7 V (**1′**). The large oxidative peak (cut in all graphs for clarity) corresponds to the oxygen evolution reaction (OER) and is caused by significant anodic polarization of the electrodes in water. However, the nature of the main reductive peak remains unclear. To properly assign this peak, the carbon cloth is subjected to the same electrochemical treatment without taurine in the electrolyte as a control sample (see Figure 1b). Here, an oxidative shoulder around 1.3 V (**2**) is visible and this peak has been reported to be coupled to the reduction peak observed around −0.5 V (**2′**) and may arise from quinone–hydroquinone type of transformations on the carbon surface.^53,54^ This peak pair is also present when the GCE is cycled in the PBS electrolyte (Figure 1d). The most prominent reduction peak in Figure 1b is around 0.7 V (**3′**) and can be attributed to the reduction of adsorbed molecular oxygen. This peak is not observed in other CV plots; the absence in GCEs (Figure 1c,d) can be explained by the fact that significant oxygen is not evolved with small-surface-area electrodes, so its subsequent reduction is not visible. However, the absence of this reduction peak (**3′**) in Figure 1a highlights that the oxidation of taurine competes with the oxidation of water or that the resulting coating prevents the reduction of molecular oxygen. Then, also considering the absence of quinone peaks (**2–2′**), the main reduction peak (**1′**) in Figure 1a,c can be attributed to the electrochemistry of taurine that is not necessarily related to the electrografting reaction. The small shoulder in Figure 1a,c around 1 V (**1**) can be attributed to the oxidation of the amine moiety of taurine. There is an additional reduction peak (**4′**) in Figure 1d, which may correspond to the reduction of molecular oxygen on the GCE, but its exact nature is not important for this work.

Since the anodic potentials may functionalize the surface with catalytically active oxide species,^55,56^ it is important to separate the influence of the electrochemical protocol from the influence of taurine on electrode properties. Thus, we applied the treatment conditions described above to all carbon electrodes for the rest of the study. Electrodes subjected to cyclic potential treatment in 0.1 M taurine and 0.1 M PBS are named **Taurine-treated GCE** and **Taurine-treated cloth** for the GCE and porous carbon cloth, respectively. The electrodes that were subjected to the same potential treatment in 0.1 M PBS solution without any taurine are named **e-treated GCE** and **e-treated cloth** for GCE and porous carbon cloth, respectively (e-treated stands for electrochemically treated). We also studied the influence of cycle number on the degree of electrografting and found that the reaction started to level off around 20 cycles (Figure S1a). However, the cyclic treatment in PBS features an ever-increasing current with increasing cycle number (Figure S1b), suggesting that oxidation of carbon fibers is not self-limiting, possibly with an accompanying mass loss.^57^ The polarization performance of electrodes with extended treatment conditions can be found in Figure S2.

**Figure 2:**
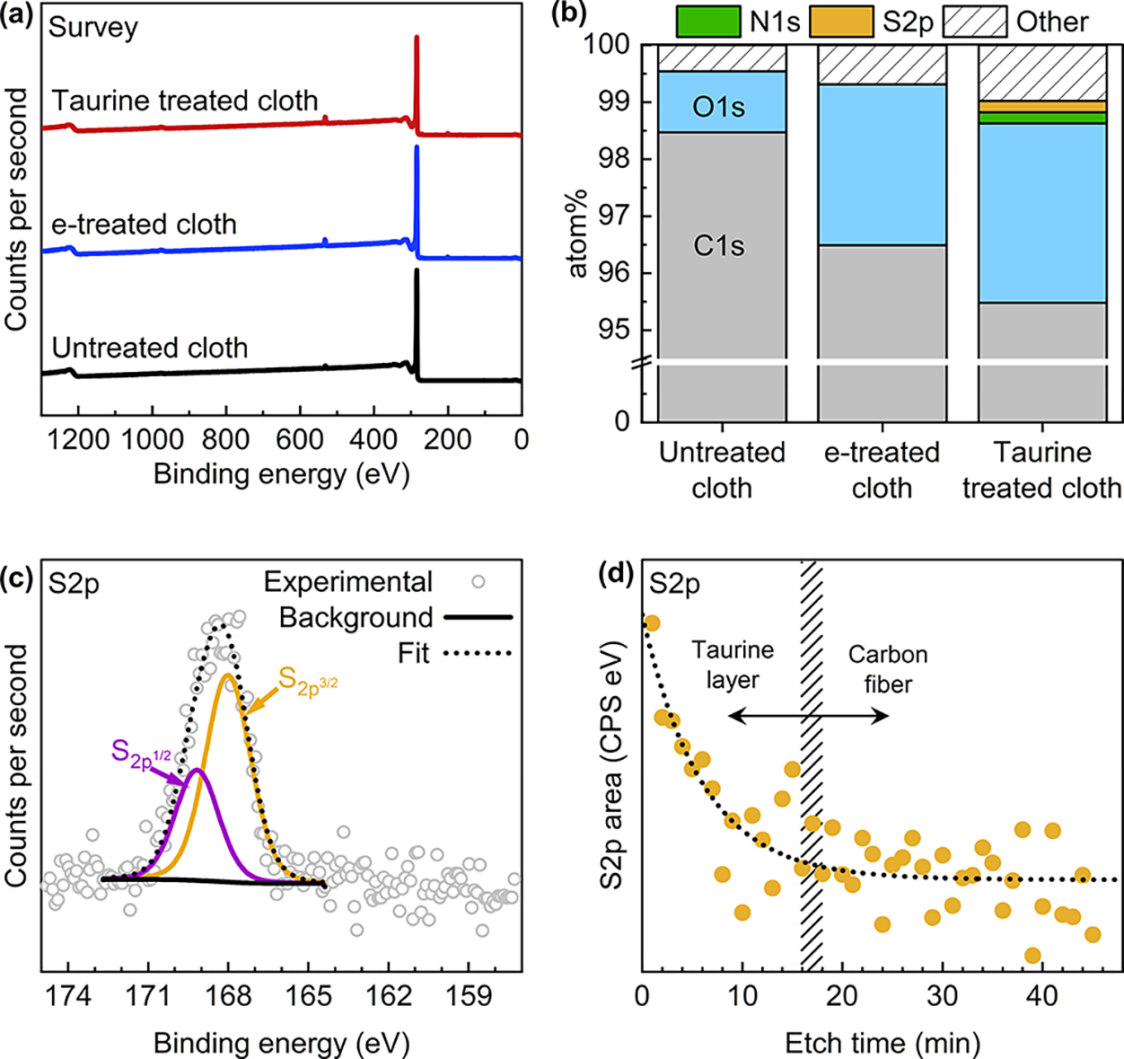
XPS of the electrode surfaces to quantify the functional groups. (a) XPS survey spectra of untreated, e-treated, and taurine-treated cloth electrodes, (b) elemental compositions extracted from the survey spectra, (c) S 2p deconvolution of the taurine-treated cloth electrode, and (d) S 2p peak area as a function of etching time. Etching is performed at 1 keV with a monochromatic Ar^+^ ion gun within the XPS chamber.

Investigation of the CV profiles suggests that taurine is oxidized, but this does not prove that it forms a coating on the surface; thus spectroscopic investigation is necessary. We elect to perform X-ray photoelectron spectroscopy (XPS) as it can resolve the chemical composition at the electrode surface. Survey spectra of cloth electrodes can be seen in Figure 2a, and the resulting elemental distribution in Figure 2b shows quantitative sulfur and nitrogen signals for the taurine-treated electrode, which are not present in the pristine or electrochemically treated electrode, indicating the presence of a taurine coating. The cloth electrode contains low oxygen content in its pristine form (99 at. % carbon), and treatments increase the oxygen content by 1.8% for the e-treated and 2.1% for the taurine-treated cloth electrode. The presence of nitrogen and sulfur in near-stoichiometric amounts (1:1) matches the stoichiometry of these atoms in the taurine molecule, further supporting that taurine is coated on the substrate. The high-resolution spectra of the S 2p region of the taurine-treated cloth (Figure 2c) can be fitted with two peaks where the S 2p_3/2_ and S 2p_1/2_ doublet are both shifted toward higher energies (∼168 eV) as expected from oxidized sulfur groups such as sulfonates.^58,59^ The presence of sulfur-containing groups upon taurine treatment has been observed previously.^41^ The N1s region of the taurine-treated cloth (Figure S3) is not well resolved, but the energy range of the peak coincides with amine moieties.^35,60,61^ Although unlikely, physisorbed species may stay on the surface of electrodes even after extensive washing, which could mask our results. To further investigate this hypothesis, the taurine-treated sample was sonicated in ultrapure water prior to performing additional XPS measurements. As seen in the high-resolution S 2p signal (Figure S4a) and N 1s signal (Figure S4b) of the sonicated electrode, the nature of sulfur and nitrogen species is nearly identical between sonicated and unsonicated samples, supporting that the taurine layer is covalently bound and is difficult to remove from the surface. For the treated electrodes, an additional Cl 2p signal is visible and may result from the association of chlorine in the PBS electrolyte to the surface carboxylic acid groups^62^ or in the case of the taurine-treated electrode also to protonated surface amines.^63^ Deconvolution of the C 1s signal in Figure S5 and distribution of major oxide groups in Figure S6 reveal a tendency to form higher oxides (carbonyl and carboxylic acid) during both treatments which are beneficial to the redox kinetics of oxide-sensitive inner-sphere redox couples.^13^ Aiming to determine the grafted layer thickness, we perform XPS depth profiling. The S 2p signal is measured after exposure to an Ar^+^ beam at a 1 keV energy for 1 min intervals, and the total area under the S 2p peak is plotted against the etching time in Figure 2d. The high-resolution spectra of the S 2p region before and after etching can be found in Figure S7. The area of the S 2p signal drops to baseline values after 16–18 min, suggesting that the carbon fiber surface is reached. Although there are no reports on the etching rate of PAN-based carbon fibers, pitch-based carbon fibers have been studied by depth profiling and scanning electron microscopy, and the authors found a dependency of tensile strength on the etching rate.^64^ Based on this finding and assuming an average tensile strength of 3.2 GPa for commercial carbon fibers,^65^ we compute an etching rate of approximately 1 nm min^–1^, which would suggest that taurine forms a layer of approximately 15–20 nm thick on the carbon fibers during electrografting.

### Electrochemical Kinetics on Model Electrodes

The effect of a coating on the electrochemical activity of the electrode can be hard to decipher as effects such as surface roughness, kinetic activity, wettability, and mass transfer are highly coupled. Decoupling the kinetic phenomena from mass transfer phenomena is especially difficult in a flow cell setting as complex flow characteristics of carbon fiber-based electrodes complicate the analysis.^66^ GCEs, with their well-defined surface area and the ease of conducting hydrodynamic studies, offer an attractive alternative to carbon fiber materials to perform fundamental analysis. It should be mentioned that although both forms of carbon are nongraphitizable,^67^ surface properties of PAN-based carbon fibers and glassy carbon differ substantially due to variation in temperature treatment and precursor materials which may play a role in the degree of functionalization during taurine treatment. Understanding this limitation, the GCE offers a strong model platform to study fundamental kinetics and to correlate the coating chemistry with the redox activity. Thus, we investigated the activity of the treated electrodes in a RDE setup for the reduction of Fe^3+^ (0.2 M) in an acidic medium (2 M HCl) that simulates the reaction conditions of the flow cell setup.

**Figure 3:**
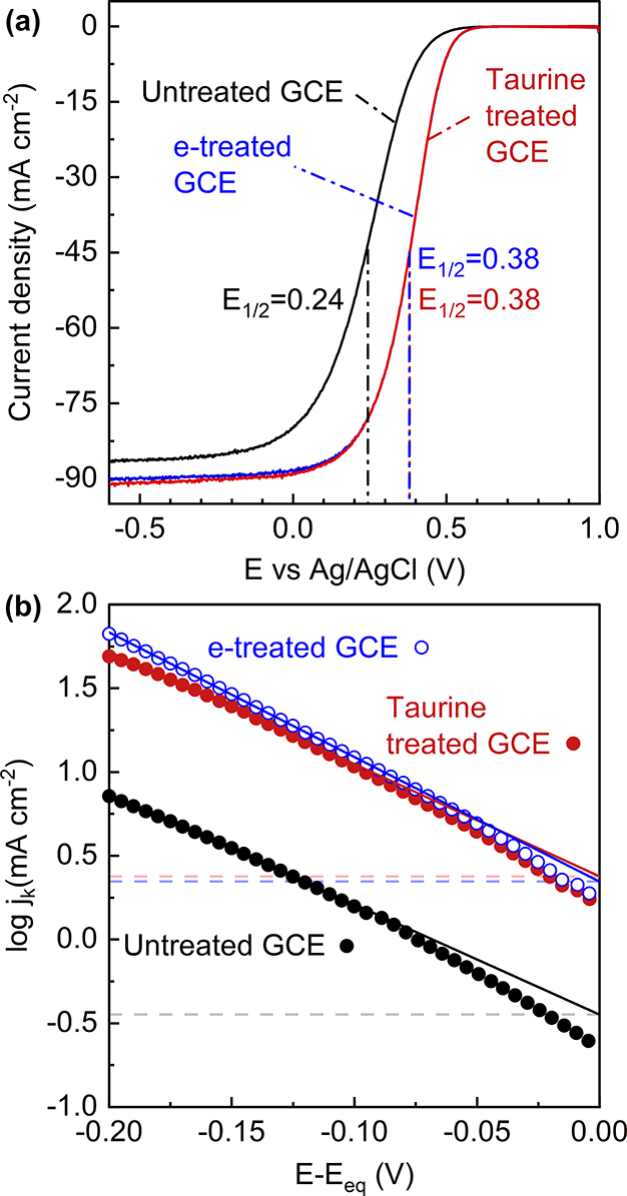
Voltammetry studies on model GCEs to assess the electrochemical rate constants. (a) Linear-sweep voltammograms at 1600 rpm and (b) mass transfer-corrected Tafel plots of untreated (black filled circles), e-treated (blue open circles), and taurine-treated GCEs (red filled circles) for Fe^3+^ reduction. The lines are the linear fit of the Tafel plots where the kinetic current is taken at the intercept at zero overpotential to calculate the charge transfer rate constants.

The LSV curves of untreated, e-treated, and taurine-treated GCEs can be seen in Figure 3a. The electron transfer rate constant of Fe^3+^ reduction on the untreated electrode is 1.84 × 10^–5^ cm s^–1^ which is on the same order of magnitude as previously reported values.^68,69^ Upon treatment, the rate constant increases by an order of magnitude, as expected due to the increased oxygen content measured in treated cloth electrodes and the sensitivity of inner-sphere Fe^3+^ on surface oxygen content.^12,13^ The difference between e-treated and taurine-treated GCEs is subtle in terms of limiting current and half-wave potential, as observed in linear-sweep voltammograms. This is quantitatively evidenced in Tafel slopes in Figure 3b, Koutecký–Levich plots in Figure S8, and geometric exchange current densities in Table 1. There is a minor improvement in the kinetic rate of Fe^3+^ reduction by taurine treatment compared to electrochemical oxidation. However, the electrochemical surface area (ECSA) and roughness (see Figure S9 for capacitance plots of GCEs) of the e-treated GCE are more than double those of the taurine-treated GCE, which suggests that improvements in the electrochemically oxidized sample may partly arise from the increased surface area. The catalytic effect of the taurine coating becomes apparent when the exchange current densities are normalized to the ECSA instead of geometric surface area as shown by the *J*_0_^ECSA^ values in Table 1. The lower roughness of the taurine-treated sample may result from an oligomeric/polymeric coating forming on the GCE and/or protection from etching of the electrode surface during high anodic potentials. The increased roughness of the electrochemically treated sample supports this argument, and the formation of a porous hydrated film has been observed before with the electrochemical activation of carbon electrodes.^57,70,71^ Consequently, taurine-treated surfaces have favorable surface chemistry for electrochemical reduction of Fe^3+^ that is not related to an increase in surface area. Another inner-sphere and oxide-sensitive redox couple, V^2+/3+^, showed higher reversibility on sulfonic acid-functionalized surfaces as well.^32^ It must be mentioned that although the substrates are different (GCE vs carbon cloth), kinetic activity determination by hydrodynamic voltammetry is more accurate than analysis of peak-to-peak separation for porous electrodes (a.k.a., Nicholson method) due to interference of mass transfer effects.^66,69,72^

**Table 1:** ECSAs, Roughness Values, Charge Transfer Rate Constants, Current Densities, and ECSA-Normalized Charge Transfer Rate Constants of Untreated, e-Treated, and Taurine-Treated GCEs

sample	ECSA (cm^2^)	*r*	10^–5^*k*^0^ (cm s^–1^)	*J*_0_^geo^ (mA cm^–2^)	*J*_0_^ECSA^ (mA cm^–2^)
untreated GCE	0.15	2.18	1.84	0.35	0.46
e-treated GCE	0.61	8.77	11.5	2.2	0.71
taurine-treated GCE	0.25	3.65	12.3	2.4	1.9

### Redox Flow Cell Performance

**Figure 4:**
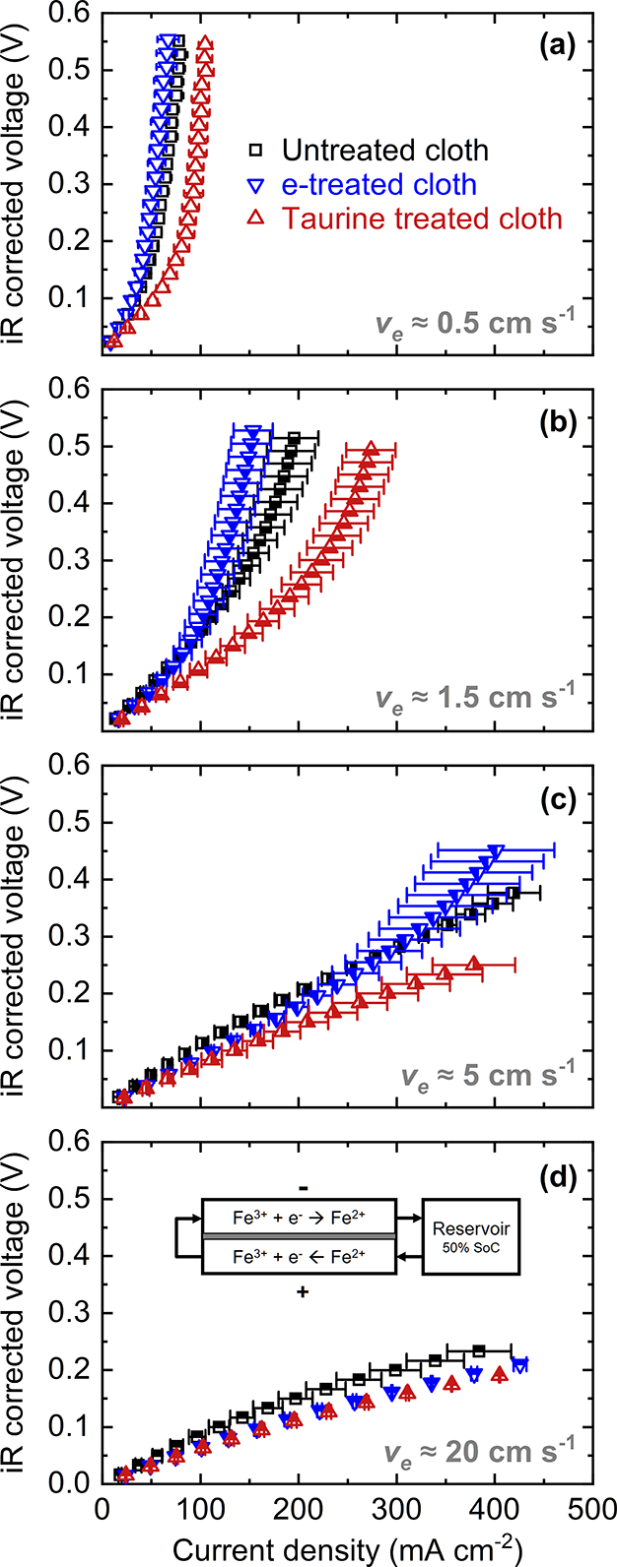
Redox flow cell experiments in the single-electrolyte configuration to characterize the performance of porous electrodes in operando. (a) Polarization curves of untreated, e-treated, and taurine-treated cloth electrodes at 0.5, (b) 1.5, (c) 5, and (d) 20 cm s^–1^. The electrolyte is 50% state-of-charge Fe^2+/3+^ (0.2 M total) in hydrochloric acid (2 M HCl). A schematic of the single-electrolyte flow cell setup employed in this study is shown in the inset (d). Each curve is averaged over two separate cells. Error bars reflect the standard error of these measurements.

After investigating the chemical composition of the electrode surface and its resulting effects on electrochemical kinetics, we now investigate the performance of the taurine-treated cloth electrode in redox flow cells. The symmetric cell setup consists of two cloth electrodes sandwiching a cation exchange membrane housed between two graphite flow-through flow fields, all secured within a polyethylene body (see Materials and Methods for further details on the flow cell). We employ single-electrolyte flow cells with 50% state-of-charge Fe^2+/3+^ (0.2 M total) in an acid (2 M HCl) electrolyte to isolate the performance of the electrodes from common RFB issues such as species crossover and state-of-charge drifts.^9^ Although the kinetic activities of taurine-treated and e-treated GCEs are comparable, the flow cell tests with treated and untreated cloth electrodes feature notable differences in performance. Polarization curves of untreated, e-treated, and taurine-treated cloth electrodes at four different flow rates can be seen in Figure 4. We report superficial electrolyte velocities instead of flow rates to normalize for the influence of the flow fields and the cell geometry. For the flow cell experiments, taurine-treated cloth outperforms the untreated and e-treated cloth at all velocities and shows a significant improvement especially at low velocities, where mass transfer effects are more pronounced. At low velocities and high overpotentials, the system is limited by mass transfer and the influence of wettability, porosity, and tortuosity plays a major role. Since the electrodes in this study are microstructurally identical, we can selectively probe the wettability of the electrodes. The untreated cloth is slightly hydrophobic, which limits its performance when aqueous electrolytes are employed, and this can explain its significantly lower performance than taurine-treated cloth at lower velocities. Unexpectedly, the electrochemical treatment seems to worsen the performance at low velocities (0.5 and 1.5 cm s^–1^), even with respect to the untreated electrode, which suggests that this electrode is limited by mass transfer overpotentials. Oxidized electrodes show higher activity for the Fe^2+/3+^ redox reaction as demonstrated by RDE studies in this work and the literature,^12,13,73,74^ so we expect to see an improvement with respect to untreated cloth for both treated electrodes, and the lower slope of treated electrodes at a higher velocity (20 cm s^–1^) supports this hypothesis. At the highest velocities, mass transfer effects are largely suppressed and the cell performance is limited by kinetic overpotentials. Here, the reaction rate plays a major role in the polarization performance and the e-treated electrode catches up, featuring similar area-specific resistance as the taurine-treated electrode.

**Figure 5:**
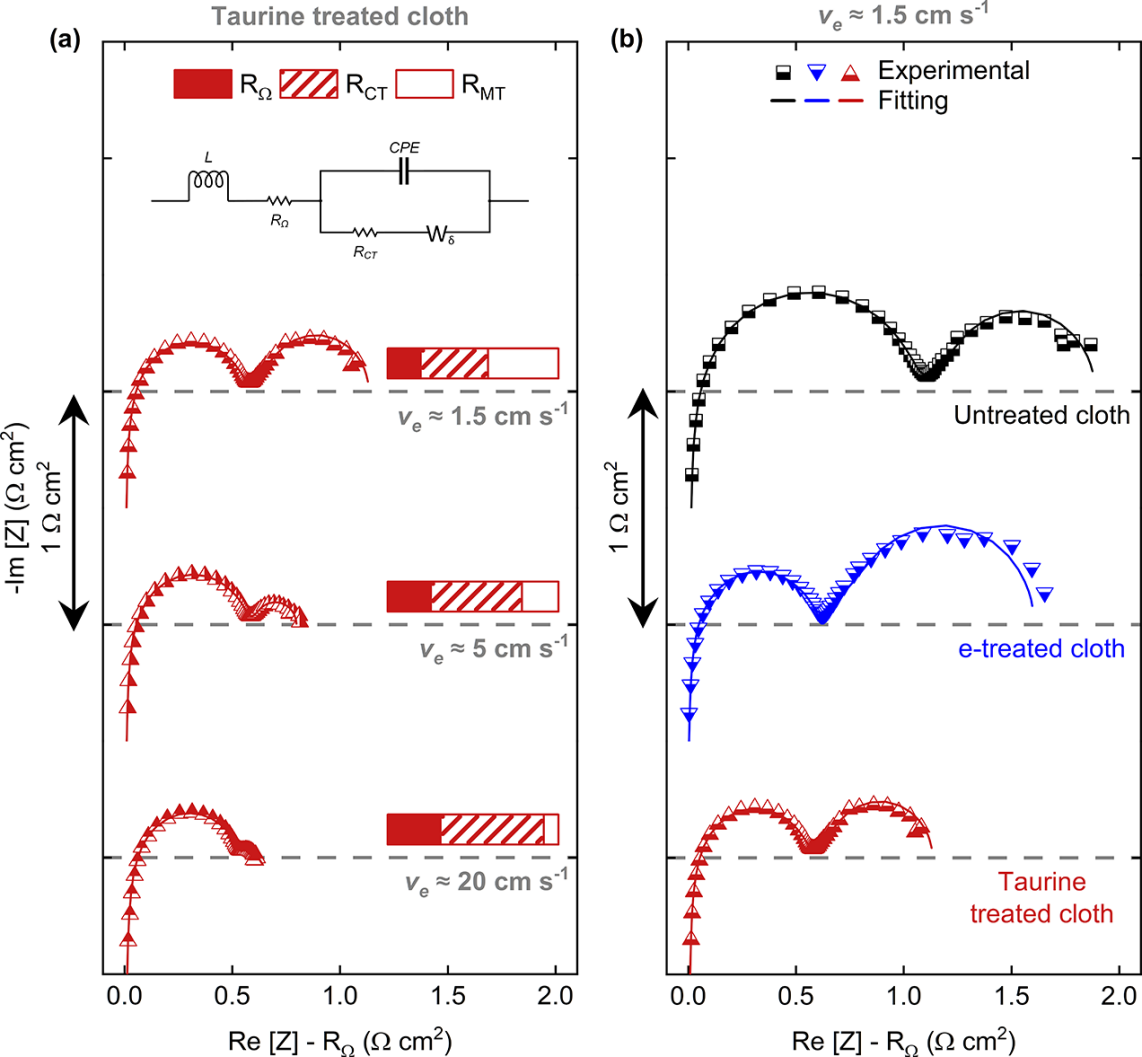
Nyquist plots of electrochemical impedance spectroscopy to elucidate resistive losses in redox flow cells. (a) Equivalent circuit diagram used in this work and impedance spectra of taurine-treated cloth electrode at three different electrolyte velocities. The bar plots at the insets show the distribution of resistances at each velocity. (b) Spectra of untreated, e-treated, and taurine-treated cloth electrodes at 1.5 cm s^–1^.

Deconvolution of kinetic, ohmic, and mass transfer resistances can reveal more information about the polarization performance of the electrodes; thus, we performed electrochemical impedance spectroscopy measurements at the OCV and various velocities. Impedance spectra of the cloth electrodes are fitted with the given equivalent circuit model,^48^ and the contribution of the resistances is presented in Figure 5. The ohmic resistance  mainly results from ionic conductivity of the membrane and electrolyte and from the electronic conductivity of the electrodes, contact resistances, and the circuitry. The charge transfer resistance  arises from redox reactions coupled to the charge transfer on the electrode surface and is sensitive to the rate of the redox reaction, which in turn is determined by the active surface area, temperature, and kinetic activity of the electrode. Finally, the depletion of active species on the electrode surface gives rise to mass transfer resistance . Aqueous RFBs have relatively low ohmic resistance, thanks to the high conductivity of ion-exchange membranes and high solubility and mobility of supporting electrolytes in water.^75^ However, metal-based redox couples in aqueous solutions feature moderate reaction kinetics which limit RFB performance.^76^

The distribution of resistances at high velocities is dominated by charge transfer and ohmic losses as forced convection minimizes mass transfer losses as seen in Figure 5a. For the taurine-treated electrode, the charge transfer resistance is dominant at all velocities, which is due to the facile mass transfer properties of the cloth electrode and taurine treatment but also due to the relatively small surface area of the cloth electrode.^48^ Investigation of impedance spectra of electrodes at low velocities (1.5 cm s^–1^) in Figure 5b reveals a decrease in charge transfer resistance of the treated cloth electrodes. Since charge transfer resistance is directly correlated with activity, this finding is in line with the increased electron transfer rate constant of treated GCEs observed in the RDE tests. Surprisingly, the magnitude of increase in ECSA observed in GCEs is not replicated here (see Figure S9 for capacitance plots of the cloth electrodes), suggesting that these carbon surfaces behave differently under electrochemical oxidation in terms of microstructural evolution and/or degree of oxidation. Nevertheless, there is a remarkable decrease in the mass transfer resistance of the taurine-treated electrode compared to untreated and e-treated electrodes, which can be observed more clearly in Table 2. We hypothesize that the increased wettability of the taurine-treated electrode, thanks to surface-bound sulfonic acid and amine groups, increases the performance of the cloth electrode. It has also been reported that the Coulombic attraction of the redox active species toward the electrode surface improves the performance of the system,^77^ which is a possible mechanism with surface-bound taurine molecules as its sulfonic acid groups have very low p*K*_a_. To further corroborate the findings from the flow cell studies, we have performed neutron imaging to understand the dynamic wetting behavior of taurine-treated and untreated cloth electrodes under representative operating conditions in a flow cell setup.

**Table 2:** Extracted Resistances through Equivalent Circuit Fitting and ECSA of Electrodes

sample	*R*_Ω_^a^ [Ω cm^2^]	*R*_CT_^a^ [Ω cm^2^]	*R*_MT_^a^ [Ω cm^2^]	ECSA^b^ [cm^2^]
untreated cloth	0.27 ± 0.01	0.99 ± 0.06	0.83 ± 0.01	86 ± 4
e-treated cloth	0.27 ± 0.01	0.63 ± 0.04	0.90 ± 0.10	108 ± 8
taurine-treated cloth	0.28 ± 0.01	0.60 ± 0.05	0.61 ± 0.04	105 ± 3

a Resistances of two different cells averaged at a superficial velocity of 1.5 cm s^–1^. Error is the standard deviation from the mean.

b ECSA of two cells averaged. Error is the standard deviation from the mean.

### Neutron Imaging/Wettability

Wettability is one of the most critical properties to tackle in electrode design for aqueous RFBs as increased hydrophilicity corresponds to the increased contact area between liquid and solid phases and consequently to increased overall current density. Unfortunately, probing the wettability of porous electrodes is difficult with conventional diagnostic techniques such as contact angle measurements due to the roughness and heterogeneity of the materials.^78,79^ Imaging techniques offer a good alternative as they allow operando measurements while the wetting and nonwetting phase within the porous material can be tracked. Among the techniques used so far to image RFB electrodes, fluorescence measurements require transparent cells and cannot image the electrode through the thickness,^80^ while X-ray tomography offers high in-plane resolution but necessitates cell modifications.^81^ Neutron radiography, on the other hand, is highly sensitive to water molecules and neutrons are not attenuated strongly by most housing materials (e.g., aluminum, steel, carbon), enabling imaging under representative conditions with minimal cell modifications. More importantly, neutrons allow imaging of the entire electrode thickness and can probe the internal wettability of electrodes, offering a powerful tool to compare different electrode treatments.^82^ With the use of the tilted detector configuration to enhance the in-plane resolution at the NEUTRA beamline of Paul Scherrer Institute, we visualized the influence of taurine electrografting on electrode wettability as a function of liquid velocity.^49^

**Figure 6:**
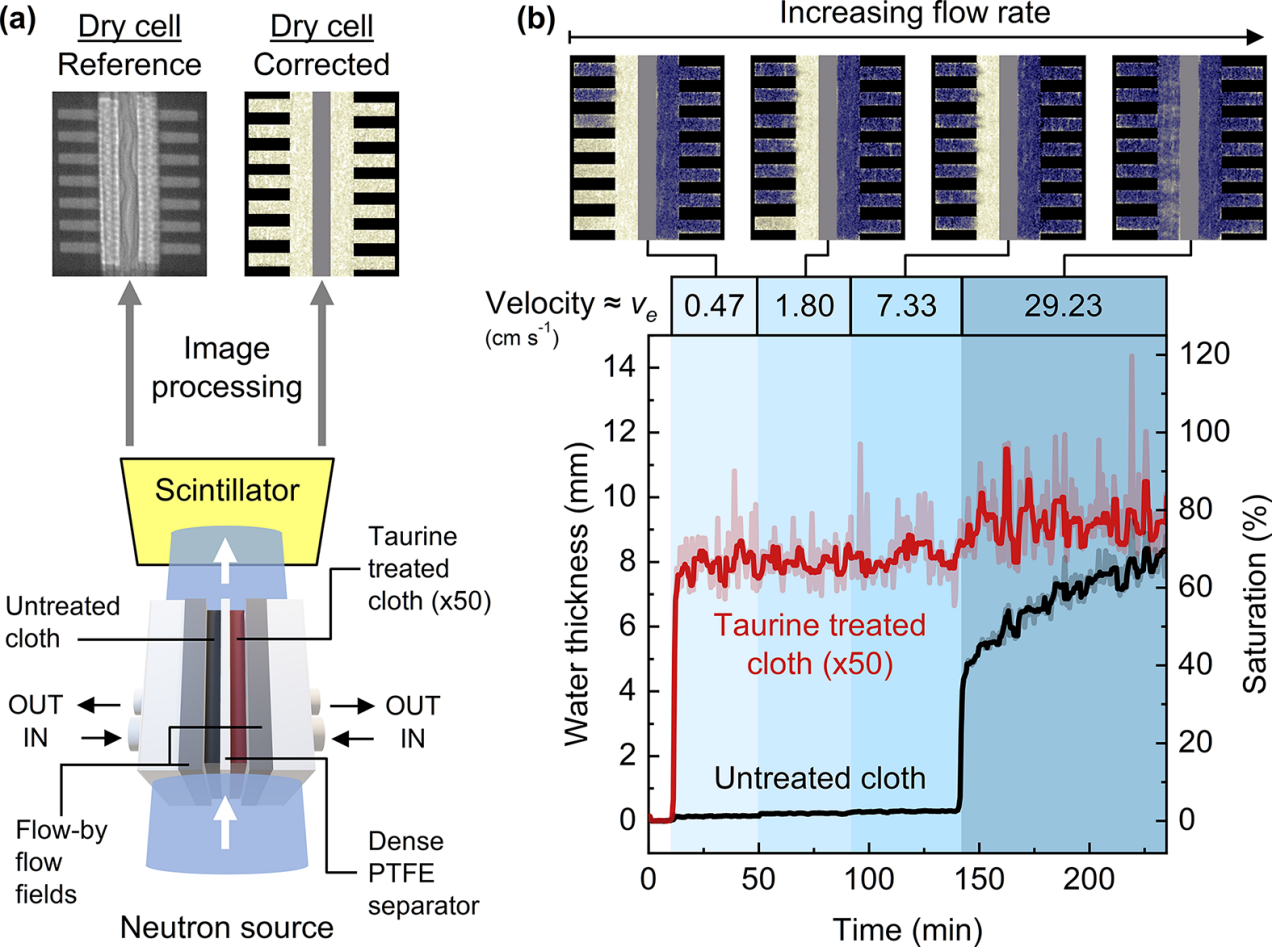
Neutron radiography experiment to visualize electrode wetting operando in a flow cell. (a) Scheme of the in-plane neutron imaging where untreated and taurine-treated (×50) electrodes, separated by a dense PTFE film, are enclosed by flow-by flow fields and plastic housing in a flow cell setup. (b) Water thickness and saturation of untreated and taurine-treated (×50) cloth electrodes over time where the electrolyte velocity in flow channels is increasing in stages. A snapshot of each velocity zone is given on top of the graph where yellow depicts dry areas and blue depicts the electrolyte.

The wettability of electrodes was investigated in a flow cell setup, where one cloth electrode is treated with taurine (placed on the left compartment) under extended cycling conditions (50 cycles) to further increase hydrophilicity and the other one is kept untreated. For comparison, we placed the untreated electrode on the right compartment, enabling direct comparisons between both electrodes at a given electrolyte velocity. The electrodes were separated by a dense PTFE separator to eliminate membrane-induced wetting and liquid crossover, and flow-by flow fields were employed to eliminate forced convection through the electrodes. With this experimental design, we study electrolyte infiltration into porous electrodes. The electrolyte solution is pumped into both compartments at step-wise increasing superficial velocities of 0.47, 1.80, 7.33, and 29.23 cm s^–1^, and images are taken in the in-plane direction throughout the experiment as seen in Figure 6a. Further details on the experimental setup and image processing can be found in Materials and Methods. A representative image of the cell under dry and wet conditions after image processing is also depicted in Figure 6a, showing the flow field channels, electrodes, and the separator. The woven pattern of the cloth electrode can be seen in the dry image. The dry cell image is used as a reference and subtracted from the images with electrolyte to remove the contribution of cell parts so that only the electrolyte can be seen through the thickness of the electrodes, as illustrated in Figure 6b. Here, we measured the water thickness and electrode saturation as a function of electrolyte velocity (in different shades of blue) and time. We find that the taurine-treated (×50) electrode instantaneously wets at the lowest velocity and reaches nearly maximum electrode saturation. Considering that the convection is not forced with this flow field design and low velocity, we hypothesize that the capillary forces, thanks to a low contact angle between the water and the electrode surfaces, are facilitating near complete wetting of the electrode. On the contrary, the wetting of the untreated electrode is dependent on the inlet pressure and the electrolyte cannot penetrate the untreated electrode for the first three velocities. When flow velocity is increased to 29.23 cm s^–1^, liquid pressure overcomes the breakthrough pressure of the untreated electrode and the pores get filled with the electrolyte. Interestingly, none of the electrodes reach 100% saturation over the course of the experiment, which we hypothesize might be due to the use of a flow-by flow field design that does not induce significant convective transport. This experiment has been transformed into a video format where the filling of electrodes over time can be found in the Supporting Information.

So far, we have demonstrated the improvements of taurine-treated electrodes for aqueous electrolytes in terms of activity and wettability. Next to these performance metrics, the stability of electrode interfaces under the aggressive electrochemical environment plays a critical role. If the interaction between the anchored functionality and the substrate is not strong enough (e.g., weak interactions as opposed to a covalent bond), the electrode functionality can be lost or negatively impacted over a lifetime.^15,56^ Thus, it is critical to assess the stability of the electrodes for prolonged operation. We performed preliminary electrode stability measurements using the single-electrolyte flow cell configuration under open-circuit potential (OCV) and applied voltage conditions (see the Supporting Information—**Stability tests**). We utilize impedance spectroscopy to monitor the variations of ohmic, charge transfer, and mass transfer resistances over 80 h. The overall resistance of the untreated electrode increases from ∼3 to ∼4.3 Ω cm^2^ after ca. 40 h. On the contrary, the taurine-treated electrodes feature a stable resistance (∼1.6 Ω cm^2^) over the entire duration of the experiment, which reveals a protective effect of the taurine layer (see Figure S10). Among the cell resistances, we find that, over time, the charge transfer resistance experiences the largest relative increase, which suggests that either the surface chemistry or the available surface area of the electrodes varies during operation. We hypothesize that the covalent bonding of taurine on carbon electrodes and its hydrophilic nature may protect the electrodes against local variations in the electrolyte available area and localized extreme currents that might induce further degradation. Although beyond the scope of this work, future studies should determine the mechanism preventing corrosion to enable the bottom-up design of protective coating layers.

## Conclusions

In this work, we have introduced a facile and versatile surface modification strategy based on electrografting of taurine to functionalize porous carbonaceous electrodes for use in RFBs. We performed a suite of spectroscopic, hydrodynamic, and electrochemical measurements to correlate surface properties of the functionalized electrodes with key performance metrics in flow batteries with an iron electrolyte. We revealed that the taurine coating increases the kinetic rate for iron (III) reduction on flat carbon surfaces by an order of magnitude (12.3 × 10^–5^ vs 1.84 × 10^–5^ cm s^–1^) while improving mass transport for porous electrodes by decreasing the mass transfer resistance by ∼25% at an electrolyte velocity of 1.5 cm s^–1^. The wetting behavior of the treated and untreated electrodes was investigated with operando neutron imaging, where spontaneous imbibition of the taurine-treated electrode has been observed, motivating its use for aqueous electrolytes and operation at lower flow rates. The flow cell results with a single iron redox couple motivate further research in electrografting as a surface modification strategy for all-iron, iron–chromium, and vanadium flow batteries. Electrografting emerges as a promising technique to design tailored and controlled functional interfaces in porous electrodes to tackle ubiquitous challenges in electrochemical reactors related to sluggish kinetics, incomplete wettability, and suppressing undesired reactions.

## Supporting Information Available

50-cycle electrografting of the GCE and cloth electrodes, polarization curves of 50-cycle cloth electrodes, additional XPS results, Koutecký–Levich plots, CV curves for capacitance measurements, stability tests of taurine-treated and untreated electrodes, SEM images of the GC plate and cloth electrodes, and pressure drop measurements (PDF)
Electrolyte penetration into untreated vs taurine-treated electrode (×50) in a flow cell setup as visualized by neutron radiography (MP4)
