# Supplementary material for: Taurine Electrografting onto Porous Electrodes Improves Redox Flow Battery Performance
Source: ACS Appl Mater Interfaces. 2022 Sep 7;14(37):41883–95. doi: 10.1021/acsami.2c08211 (PMC9501779; doi:10.1021/acsami.2c08211)
Supplement: Supplementary file 1 — am2c08211_si_001.pdf [file am2c08211_si_001.pdf]

Supporting Information

Taurine electrografting onto porous electrodes improves redox flow battery performance

Emre B. Boz[1,2], *Pierre Boillat*[3,4], *Antoni Forner-Cuenca*[1,2,]*

[1]Electrochemical Materials and Systems, Department of Chemical Engineering and Chemistry, Eindhoven University of Technology, P.O. Box 513, 5600 MB Eindhoven, The Netherlands

[2]Eindhoven Institute for Renewable Energy Systems, Eindhoven University of Technology, P.O. Box 513, 5600 MB Eindhoven, The Netherlands

[3]Electrochemistry Laboratory, Paul Scherrer Institute, Forschungsstrasse 111, CH-5232, Villigen PSI, Switzerland

[4]Laboratory for Neutron Scattering and Imaging, Paul Scherrer Institute, Forschungsstrasse 111, CH-5232, Villigen PSI, Switzerland

Corresponding Author:

*Antoni Forner-Cuenca, a.forner.cuenca@tue.nl, +31 40 247 6258

Table of Contents

Section 1 – Supporting experiments 3
Electrografting for 50 cycles 3
Figure S1 – *CV plots for 50 cycle electrografting* 3
Figure S2 – *Polarization performance of 10 cycle and 50 cycle samples* 4
X-ray photoelectron spectroscopy 5
Figure S3 – *N1s spectra of taurine treated cloth.* 5
Figure S4 – *S2p and N1s spactra of taurine treated cloth after sonication in water.* 5
Figure S5 – *C1s spectra of cloth electrodes.* 6
Figure S6 – *C1s component distributions of cloth electrodes* 6
Figure S7 – *S2p region of taurine treated cloth before and after etching* 7
Hydrodynamic voltammetry 8
Figure S8 – *Koutecký-Levich plots of glassy carbon electrodes* 8
Capacitance measurements 9
Figure S9 – *Capacitance CV curves of cloth and glassy carbon electrodes* 9
Stability tests 10
Figure S10 – *Stability tests of untreated and taurine treated cloth electrodes* 11
Figure S11 – *Post-mortem XPS analysis of electrodes after stability tests* 12
Section 2 – Additional characterization 13
Microscopic characterization 13
Figure S12 – *SEM pictures of glassy carbon plate electrodes* 14
Effect of electrochemical treatment 15
Figure S13 – *Pressure drop measurements of cloth electrodes in flow cells* 15
Figure S14 – *SEM pictures of cloth electrodes* 16

Section 1 – Supporting experiments

Electrografting for 50 cycles

Electrografting of taurine was extended to 50 cycles to investigate the bounds of the reaction and for the sake of comparison, the same extension was carried out in PBS-only electrolyte. The taurine electrografting reaction is limited to around 20 cycles whereas the reactions in PBS-only electrolyte are not limited and hint at surface degradation via oxidation. The electrografting reactions in the main manuscript were limited to 10 cycles as they provided the best improvements with respect to cycle number. 50 cycle electrografted samples demonstrate marginal performance increase compared to 10 cycle samples (see **Figure S2**) at the expense of longer treatment times and more energy spent.

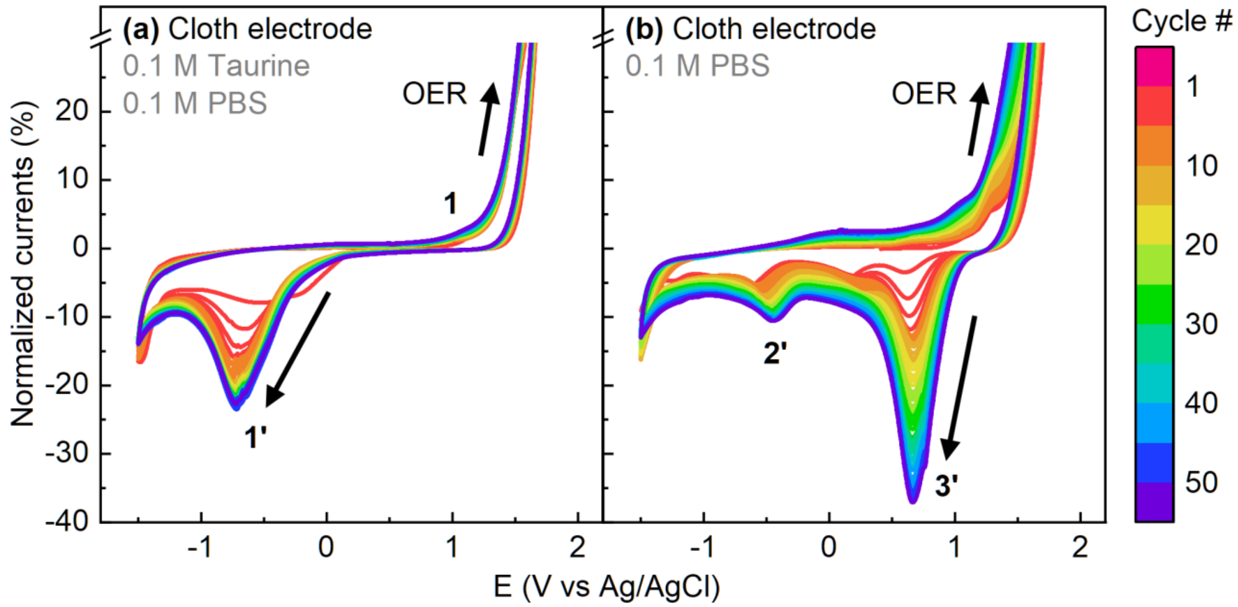

Figure S1. CV response of cloth electrodes for 50 cycles in 0.1 M Taurine + 0.1 M PBS (pH=7.4) (**a**) and only 0.1 M PBS (pH=7.4) (**b**). The peak numbers have their same significance as in main manuscript.

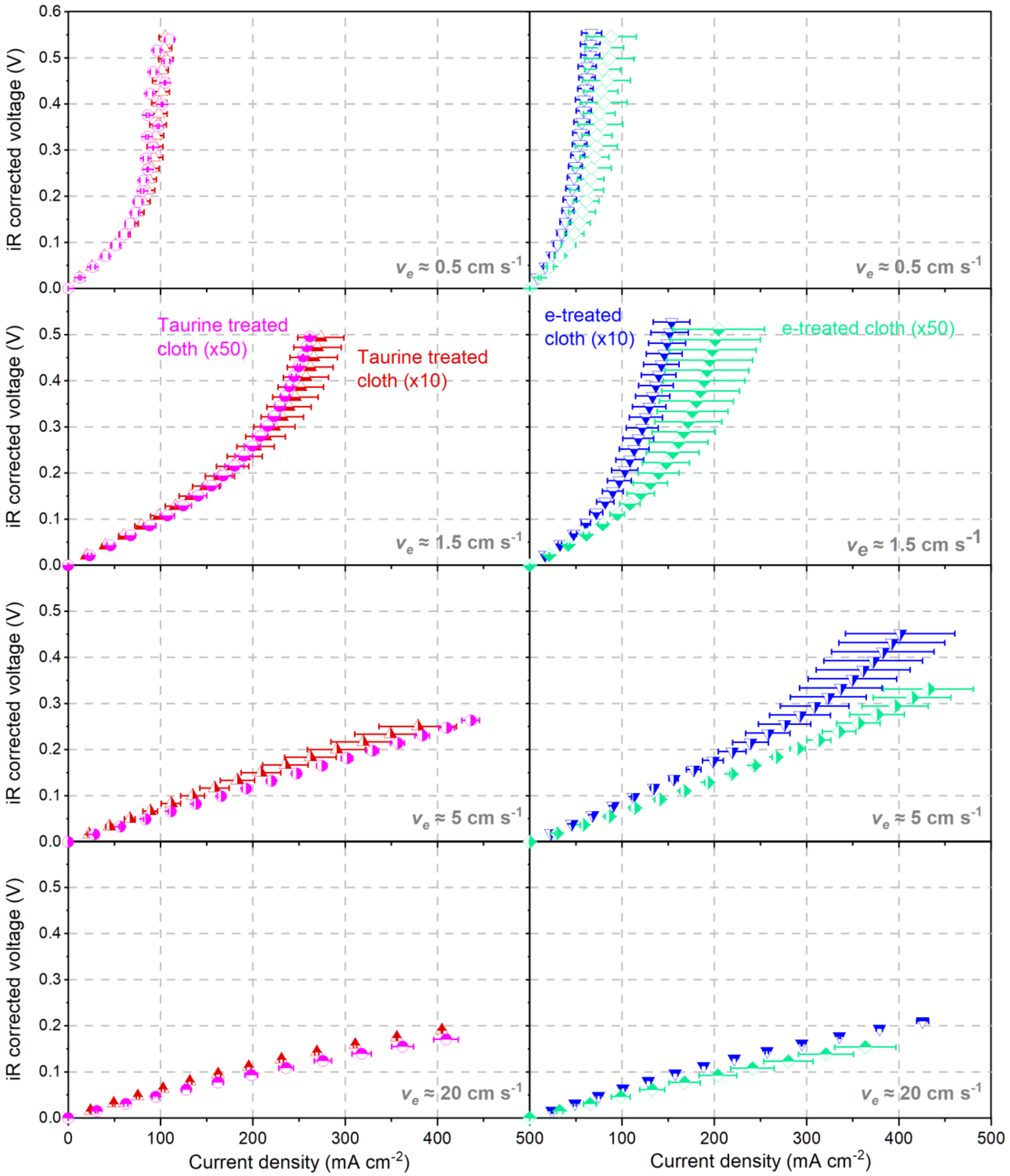

Figure S2. Flow cell polarization response of 10 cycle and 50 cycle treated electrodes compared. Polarization curves of 10 cycle and 50 cycle experiments are averaged over 2 cells. Error bars reflect standard error of these measurements.

X-ray photoelectron spectroscopy

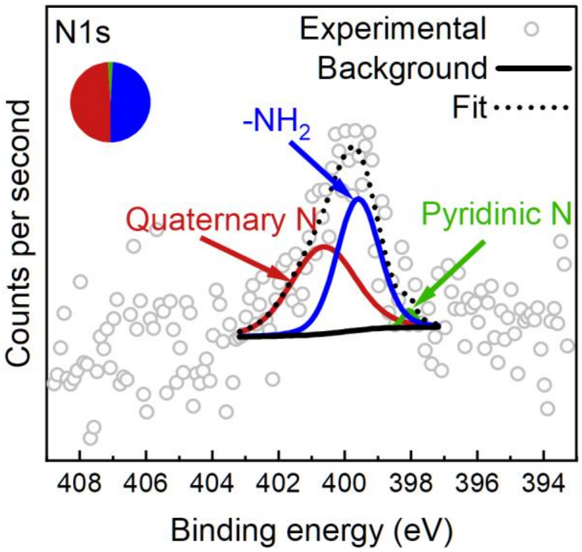

Figure S3. N1s spectra of taurine treated cloth. The bar chart inset shows the distribution of nitrogen species.

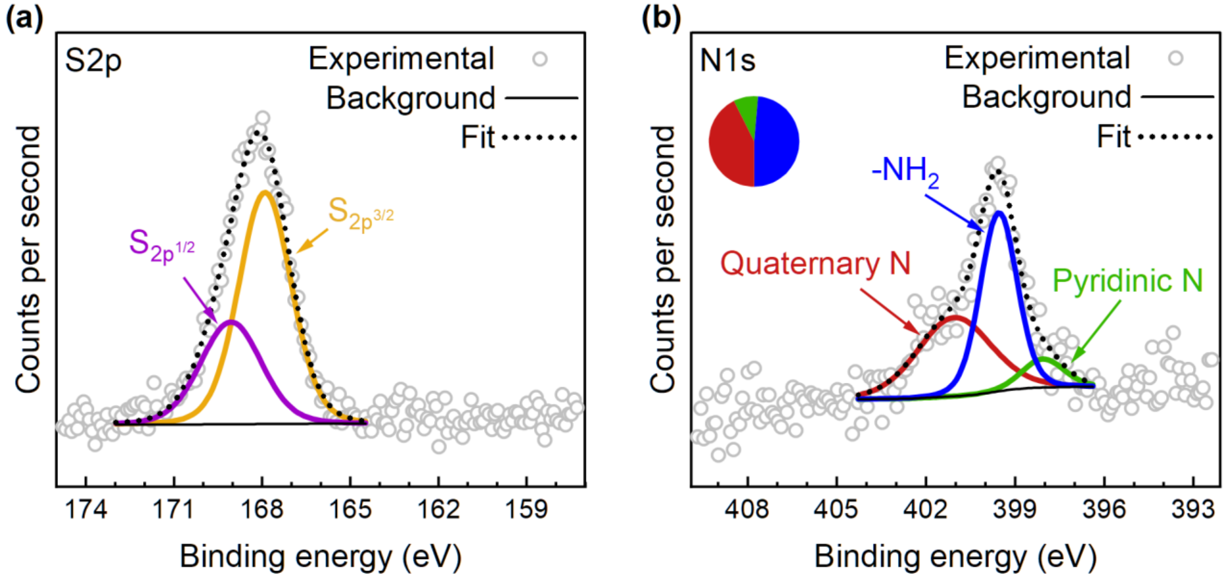

Figure S4. S2p **(a)** and N1s spectra of taurine treated cloth after sonication **(b)**. The bar chart inset in **(b)** show the distribution of nitrogen species.

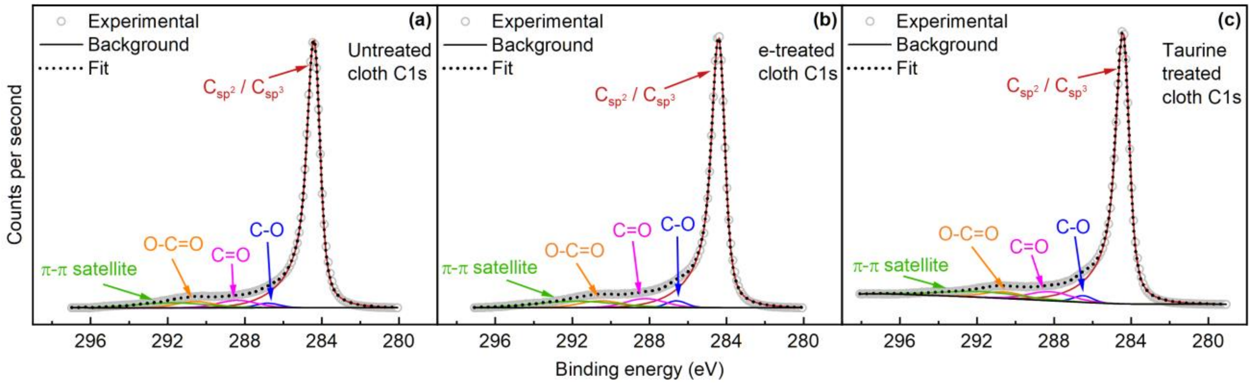

Figure S5. XPS C1s spectra and fits of untreated **(a)**, e-treated **(b)** and taurine treated cloth electrodes **(c)**.

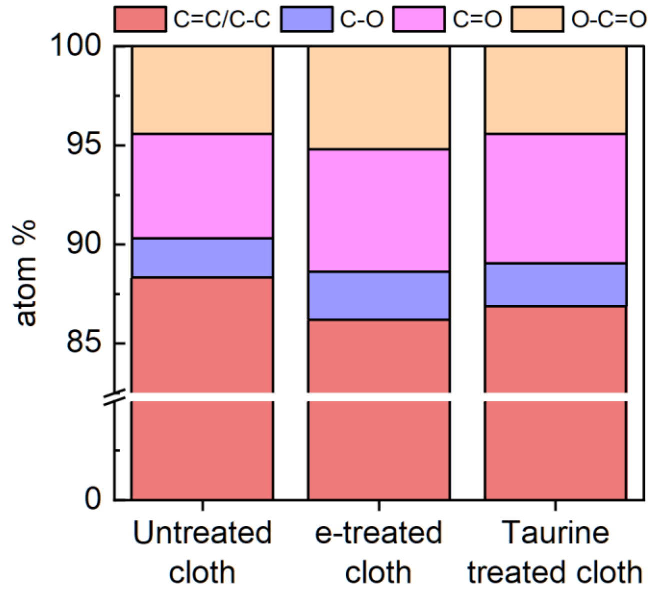

Figure S6. C1s component distributions of untreated, e-treated and taurine treated cloth electrodes based on deconvolution of C1s spectra.

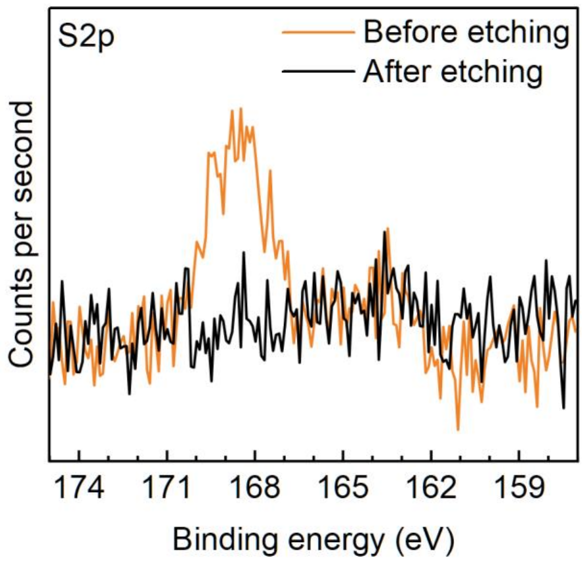

Figure S7. Raw S2p spectra of taurine treated cloth electrode before and after Ar^{+} etching. The low energy shoulder (around 163 eV) results from degradation of the sulfonate groups to other sulfur species by the high energy ion beam. The shift on the sample before etching results from proximity of the ion beam from an adjacent measurement point. Samples not exposed to the ion beam do not feature such chemical shifts.

Hydrodynamic voltammetry

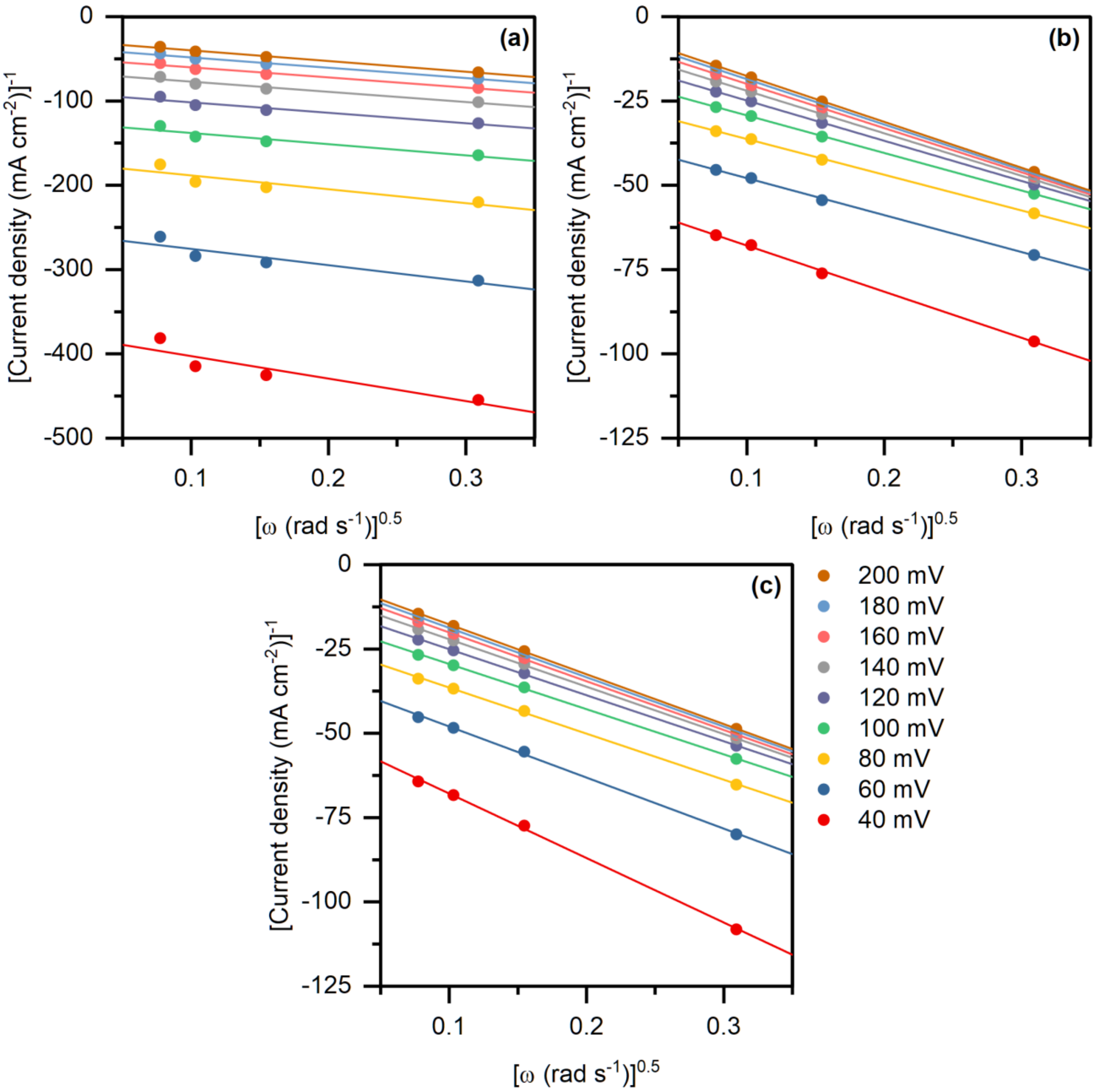

Figure S8. Koutecký-Levich plots of untreated **(a)**, e-treated **(b)** and taurine treated **(c)** glassy carbon electrodes.

Capacitance measurements

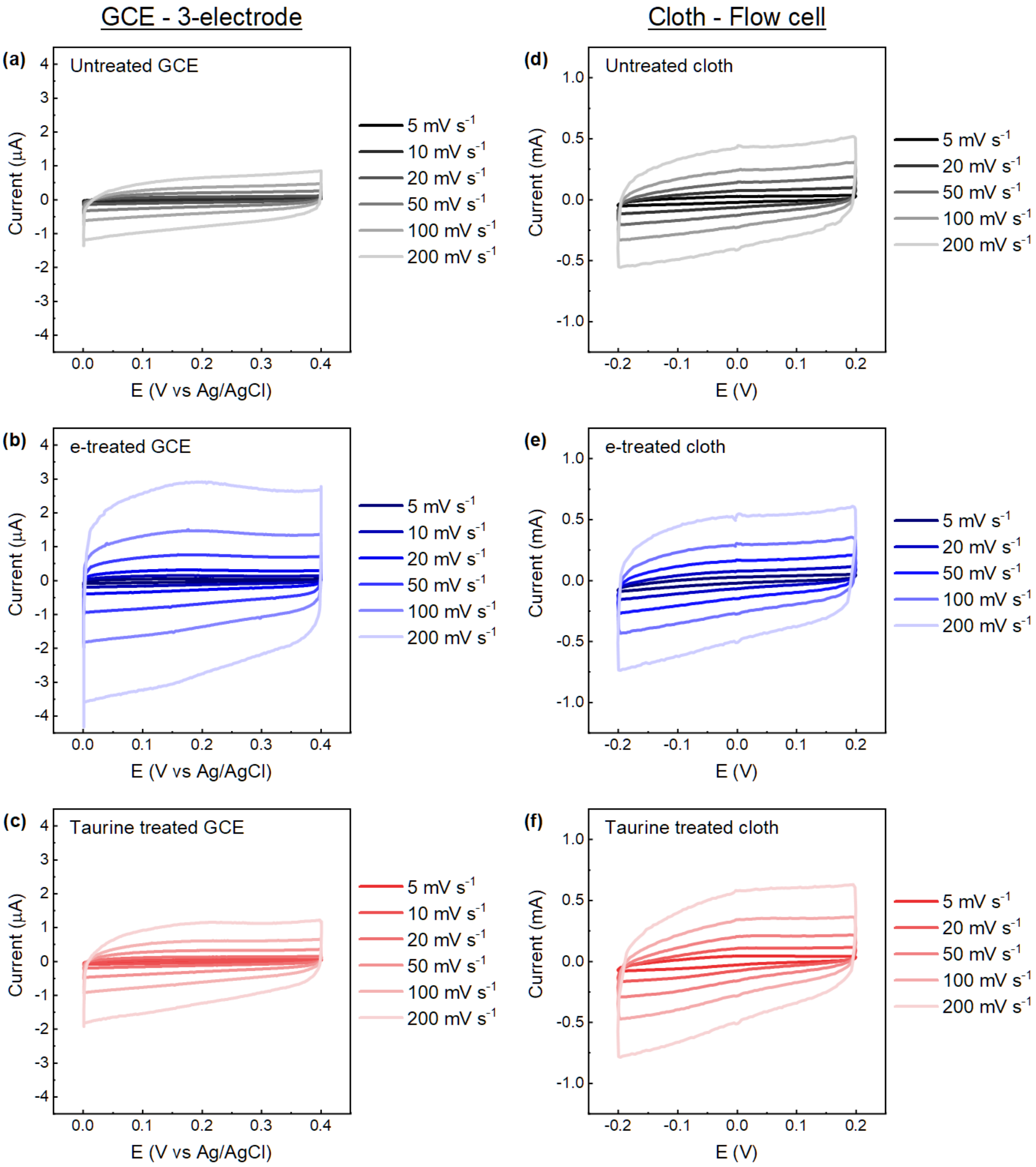

Figure S9. Cyclic voltammograms of untreated **(a)**, e-treated **(b)** and taurine treated glassy carbon electrodes **(c)** in a 3-electrode setup and untreated **(d)**, e-treated **(e)** and taurine treated cloth electrodes **(f)** in a flow cell setup for capacitance calculations.

Stability tests

The stability of taurine treated electrodes was investigated under iron electrolyte flow (0.2 M $Fe^{2+/3+}$ in 2 M HCl) with and without applied potential to understand the effects of electrolyte and prolonged cycling. The experiment flow is depicted in **Figure S10a**. Briefly; electrolyte is pumped to the cell at 5 cm s^{-1} with continuous nitrogen flow to blanket the solutions and by placing the electrolyte tank in a thermostatic bath set at 20 C° to prevent temperature induced current variations throughout the day. Impedance spectroscopy at open circuit conditions (EIS-OCV) were recorded from 200 kHz to 10 mHz. After 45 hours, the cells were subjected to a constant potential at 0.2 V (vs OCV) for 10 minutes and an EIS-OCV was performed and this sequence was repeated 50 times, taking around 17 hours. Finally, the cell is polarized to 0.4 V and previous measurement scheme is repeated. The resulting impedance spectra were fitted with the equivalent circuit model (seen in **Figure 5a**) and extracted resistances were plotted as a function of time. The entire experiment takes around 80 hours and reveals the change in the resistance of the cell in the presence of electrolyte and under applied potential.

As seen in **Figure S10b**, the resistance of untreated cloth keeps increasing during the OCV period (gray shaded area) and stabilizes around 4 Ω cm^2. Meanwhile, taurine treated electrode is remarkably stable during operation and its resistance remains stable around 1.5 Ω cm^2. After deconvolution of resistances, we can observe that significant change is occurring in the charge transfer resistance of the electrodes as seen in **Figure S10c**. Charge transfer resistance is related to exchange current density which is a function of (electrolyte accessible) area of the electrode, concentration of the active species and the kinetic rate constant for the reaction (see **Equation 1** in **Manuscript - Materials and Methods**). The concentration is kept constant by the half-cell setup employed in the study and a decrease in the surface area is not expected at these flow rates

and with the forced-convection flow through flow field design. This suggests a change in the kinetic rate constant over time, which is possible if the chemical environment of the electrode surface is altered. To understand the surface chemical state, we have performed XPS measurements on untreated electrodes post-stability (**Figure S11**), but the heavy contamination due to membrane and electrolyte species complicate the surface analysis of the electrodes and no information can be reliably obtained about the surface nature of the carbon fibers. Nevertheless, taurine treatment seems to have a protective effect on the electrode as it prevents performance degradation over the operation window.

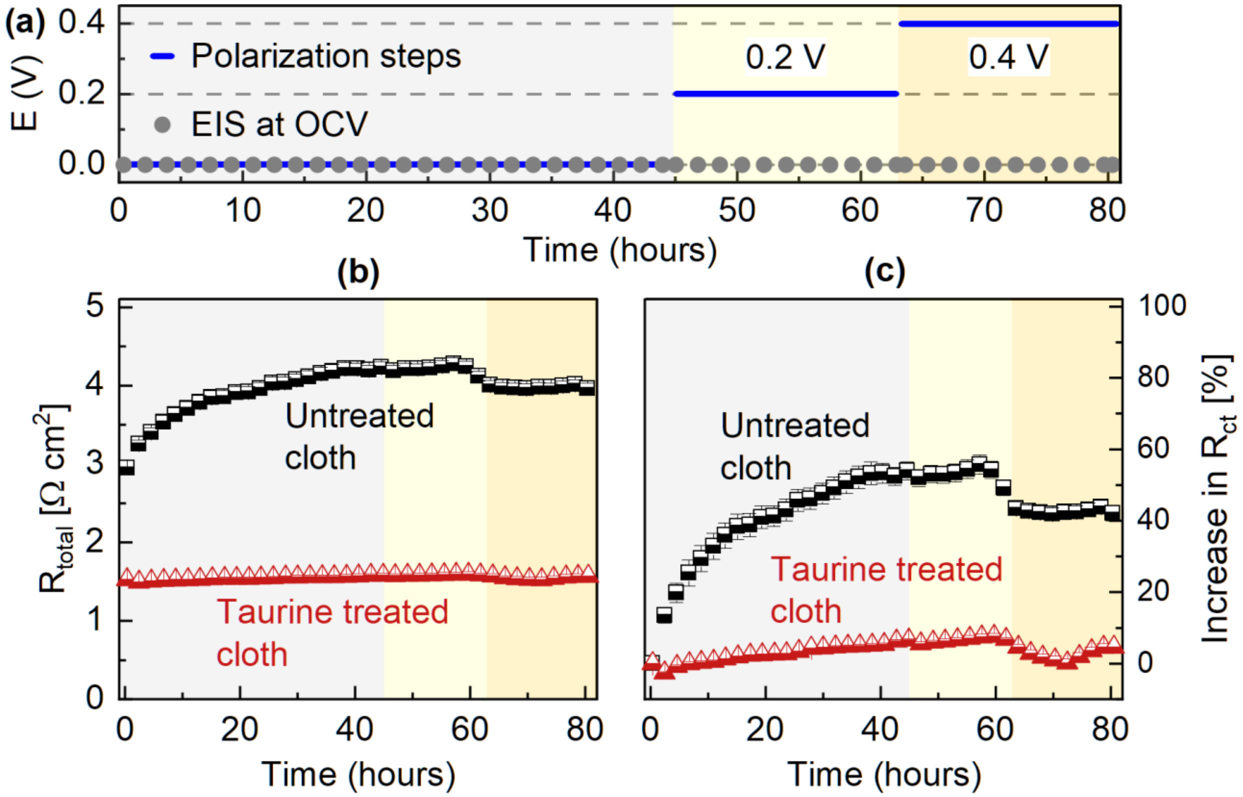

Figure S10. Experiment flow of the stability measurements **(a)**, stability of untreated and taurine treated cloth electrodes by tracking the change in total resistance with impedance spectroscopy measurements **(b)** and the contribution of charge transfer resistance to this change **(c)**. Resistances of 2 cells averaged, error bars reflect standard error.

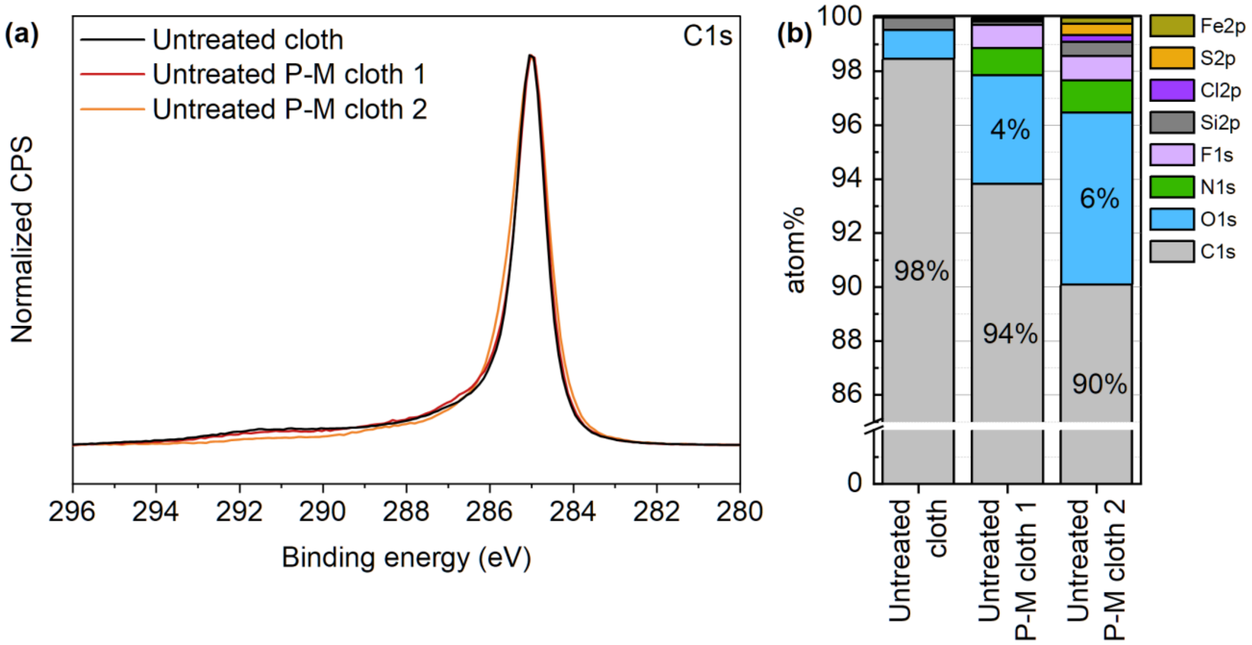

Figure S11. Energy calibrated (to 285 eV) XPS C1s high resolution spectra **(a)** and elemental composition of untreated cloth and *post-mortem* (P-M) electrodes after stability tests **(b)** where 1 and 2 are electrode numbers in one of the cells (anode/cathode identification not possible).

Section 2 – Additional characterization

Microscopic characterization

The GCE in plate form (Redox.me) has 0.5 mm nominal thickness and a surface area of 1 cm^2 on each face (top and bottom). The electrochemical treatment applied to GCE plates is the same as described in the main manuscript.

Morphological characterization of carbon cloths and GCE plates was conducted with a JEOL JSM-IT100 scanning electron microscope (SEM) at 20 kV accelerating voltage for cloth electrodes and 4 kV for GCE plates at a working distance of 10 mm. Imaging the thin coating formed by electrografting is tricky on carbon fiber substrates, so we employed coating studies on glassy carbon plates. Half of the plate was immersed in the respective electrolyte to observe the boundary between coated and uncoated part and the resulting images can be seen in **Figure S12**. In both treatments a color change is visible to the naked eye, however this could be caused by the exfoliation of the carbon flakes during oxidative treatment. In the case of taurine electrografting, a film like layer can be observed, which may suggest that there is a polymeric coating on top of the substrate. We tried to perform local energy dispersive X-ray spectroscopy on the substrate but did not obtain sufficient sulfur or nitrogen signal to indicate presence of taurine. If the layer is indeed very thin, than the penetration depth of EDX may be too much to resolve the signals coming from the top layer.

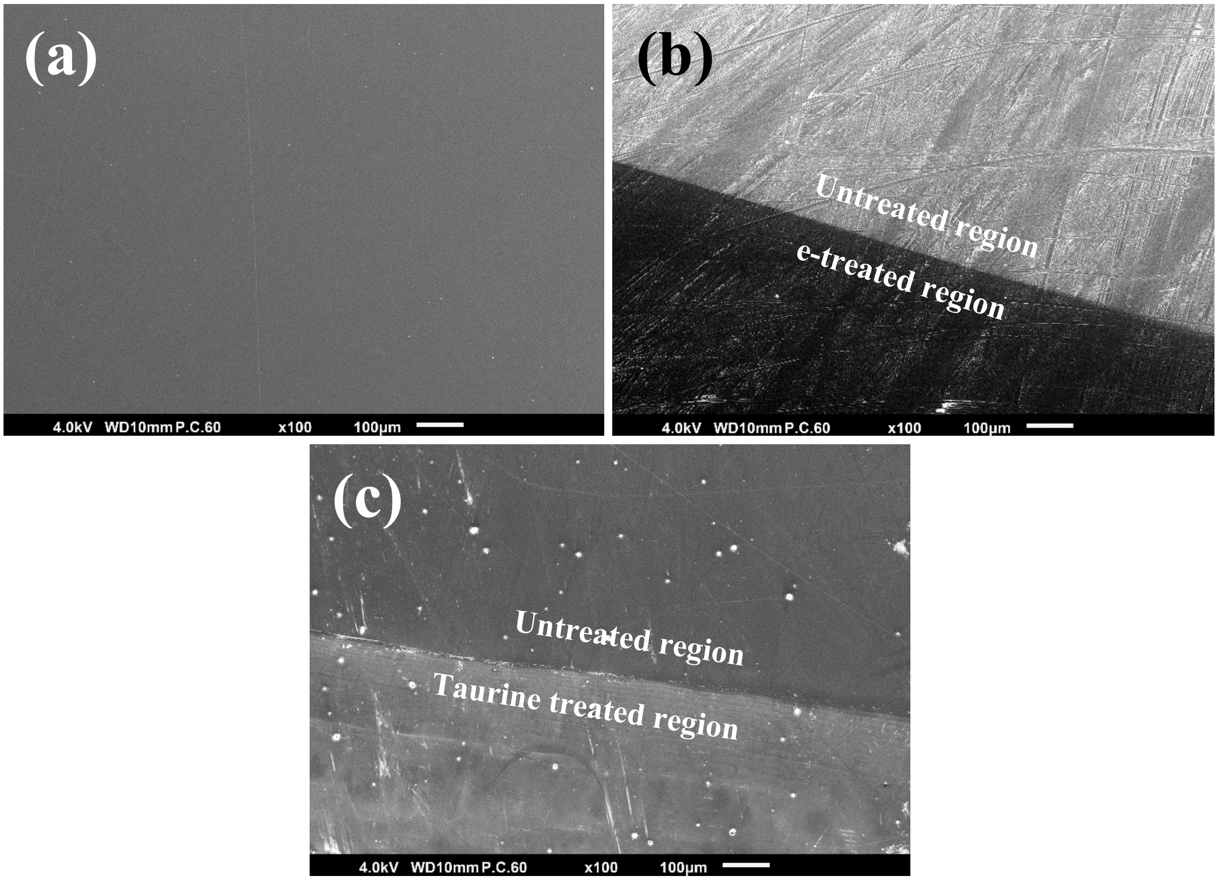

Figure S12. Untreated **(a)**, e-treated **(b)** and Taurine treated **(c)** glassy carbon plate electrodes. The border in **b** and **c** show the transition from untreated to treated region (lower parts were immersed in their respective electrolyte). The observed roughness in **b** is due to polishing and not due to treatment. The white spots in c are alumina particles from the polishing suspension.

Effect of electrochemical treatment

It is not immediately clear why e-treated cloth performs worse than untreated electrode at low flow rates. The primary reasons behind a mass-transfer limitation could be a decrease in wettability or increased hydrodynamic resistance. Wettability of e-treated cloth is expected to be high owing to its increased oxygen content (**Figure 2b**) and it was impossible to make contact angle measurements due to immediate imbibition of the droplet by the e-treated cloth. Although not an accurate measure of mass transfer resistance, pressure drop measurements (**Figure S13**) show that e-treated cloth has the lowest hydrodynamic resistance among all electrodes tested. SEM pictures of cloth electrodes also look virtually identical (**Figure S14**), which suggests there is a different reason for the low performance of e-treated cloth at low flow rates. It is also surprising that this limitation is not observed in hydrodynamic studies with GCE (see **Figure 3**), which suggests that it is either caused by the microstructure of the cloth electrode or its particular carbon chemistry.

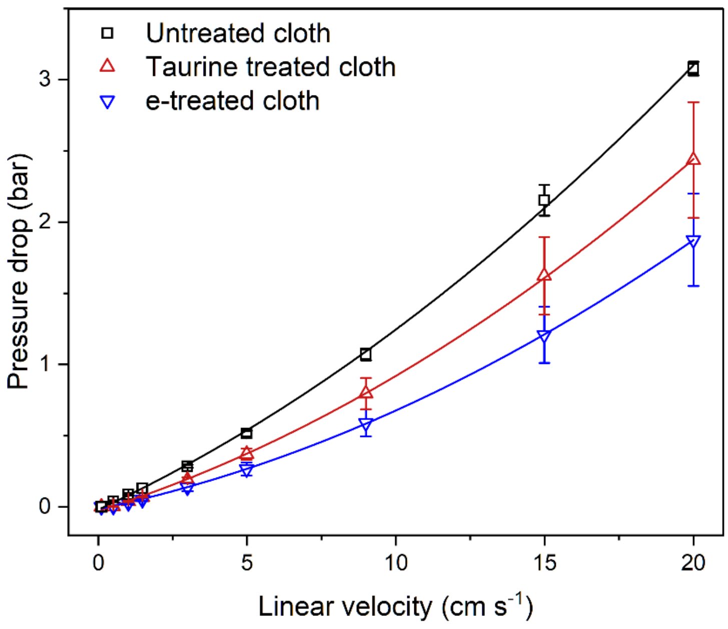

Figure S13. Pressure drop of cloth electrodes in the flow cell setup as a function of electrolyte velocity. Contribution of cell parts has not been subtracted but should be equal in all cases.

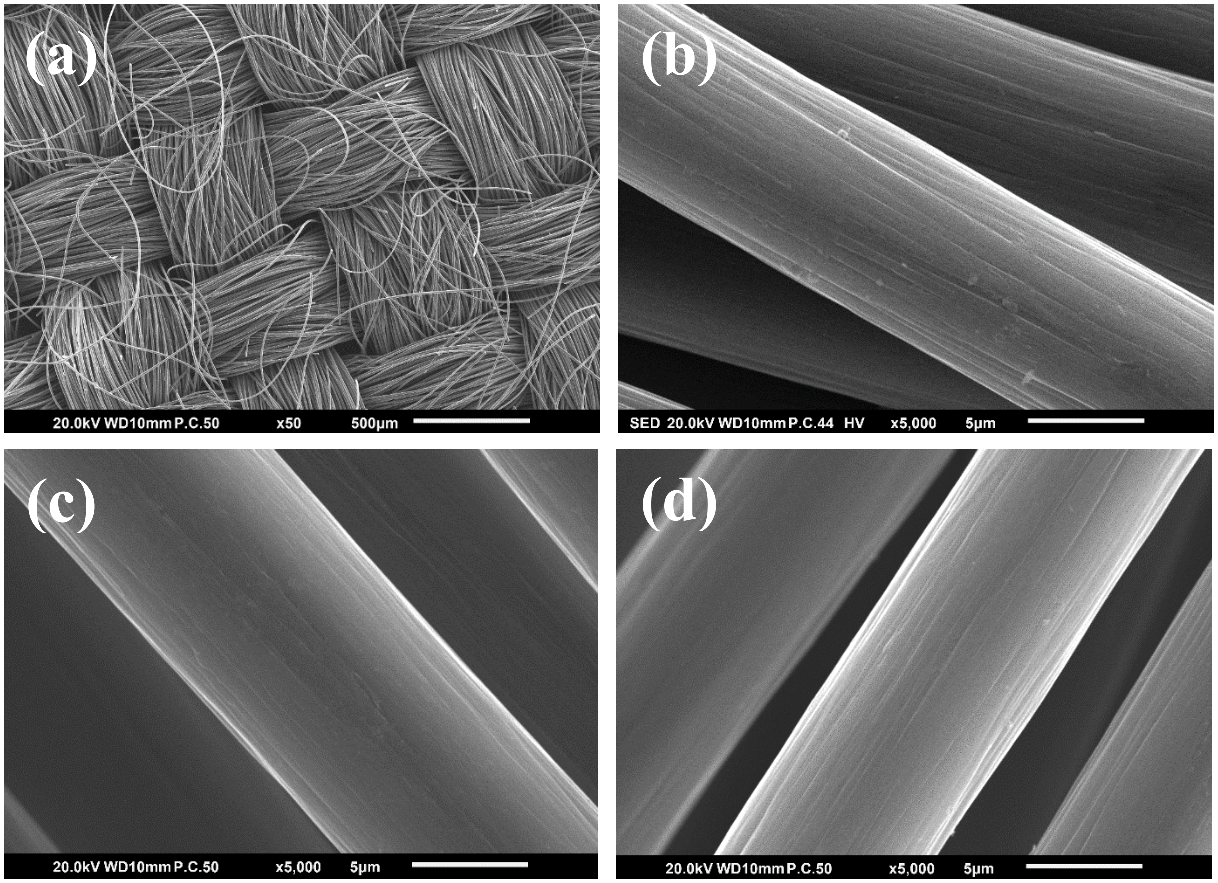

Figure S14. SEM pictures of untreated cloth electrode weave pattern (a), close-up on fibers of untreated (b), e-treated (c) and taurine treated (d) cloth electrodes.
